# Characterization of miR-122-independent propagation of HCV

**DOI:** 10.1371/journal.ppat.1006374

**Published:** 2017-05-11

**Authors:** Chikako Ono, Takasuke Fukuhara, Daisuke Motooka, Shota Nakamura, Daisuke Okuzaki, Satomi Yamamoto, Tomokazu Tamura, Hiroyuki Mori, Asuka Sato, Kentaro Uemura, Yuzy Fauzyah, Takeshi Kurihara, Takahiro Suda, Akira Nishio, Su Su Hmwe, Toru Okamoto, Tomohide Tatsumi, Tetsuo Takehara, Kazuaki Chayama, Takaji Wakita, Kazuhiko Koike, Yoshiharu Matsuura

**Affiliations:** 1 Department of Molecular Virology, Research Institute for Microbial Diseases, Osaka University, Osaka, Japan; 2 Department of Infection Metagenomics, Research Institute for Microbial Diseases, Osaka University, Osaka, Japan; 3 DNA-Chip Developmental Center for Infectious Diseases, Research Institute for Microbial Diseases, Osaka University, Osaka, Japan; 4 Department of Gastroenterology and Hepatology, Graduate School of Medicine, Osaka University, Osaka, Japan; 5 Department of Virology II, National Institute of Infectious Diseases, Tokyo, Japan; 6 Department of Medicine and Molecular Science, Hiroshima University School of Medicine, Hiroshima, Japan; 7 Department of Gastroenterology, Graduate School of Medicine, The University of Tokyo, Tokyo, Japan; University of California, San Diego, UNITED STATES

## Abstract

miR-122, a liver-specific microRNA, is one of the determinants for liver tropism of hepatitis C virus (HCV) infection. Although miR-122 is required for efficient propagation of HCV, we have previously shown that HCV replicates at a low rate in miR-122-deficient cells, suggesting that HCV-RNA is capable of propagating in an miR-122-independent manner. We herein investigated the roles of miR-122 in both the replication of HCV-RNA and the production of infectious particles by using miR-122-knockout Huh7 (Huh7-122KO) cells. A slight increase of intracellular HCV-RNA levels and infectious titers in the culture supernatants was observed in Huh7-122KO cells upon infection with HCV. Moreover, after serial passages of HCV in miR-122-knockout Huh7.5.1 cells, we obtained an adaptive mutant, HCV_122KO_, possessing G28A substitution in the 5’UTR of the HCV genotype 2a JFH1 genome, and this mutant may help to enhance replication complex formation, a possibility supported by polysome analysis. We also found the introduction of adaptive mutation around miR-122 binding site in the genotype 1b/2a chimeric virus, which originally had an adenine at the nucleotide position 29. HCV_122KO_ exhibited efficient RNA replication in miR-122-knockout cells and non-hepatic cells without exogenous expression of miR-122. Competition assay revealed that the G28A mutant was dominant in the absence of miR-122, but its effects were equivalent to those of the wild type in the presence of miR-122, suggesting that the G28A mutation does not confer an advantage for propagation in miR-122-rich hepatocytes. These observations may explain the clinical finding that the positive rate of G28A mutation was higher in miR-122-deficient PBMCs than in the patient serum, which mainly included the hepatocyte-derived virus from HCV-genotype-2a patients. These results suggest that the emergence of HCV mutants that can propagate in non-hepatic cells in an miR-122-independent manner may participate in the induction of extrahepatic manifestations in chronic hepatitis C patients.

## Author summary

A liver-specific microRNA, miR-122, is one of the key determinants of hepatitis C virus (HCV) hepatotropism and is required for efficient propagation of HCV. On the other hand, chronic infection with HCV is often associated with extrahepatic manifestations (EHMs), and a low level of HCV-RNA replication has been detected in some non-hepatic cells. Nonetheless, the detailed mechanisms underlying these phenomena remain unknown. Here, we show that miR-122 is dispensable for low-level replication or infectious particle formation, and a mutant virus adapted to miR-122-knockout cells exhibited efficient but miR-122-independent propagation. The adaptive virus of HCV genotype 2a possessed a G28A substitution in the 5’UTR and facilitated efficient replication complex formation under an miR-122-deficient condition, while it propagated at a level comparable to the wild type HCV in the presence of miR-122. Moreover, various adaptive mutations including C30U were introduced into genotype 1b, which originally had an adenine at the nucleotide position 29. These observations suggest that substitutions that yield miR-122-independent propagation are not induced during propagation in hepatocytes; however, treatment with an miR-122 inhibitor or persistent infection of HCV in non-hepatic cells may induce the emergence of mutant viruses, as evidenced by clinical samples.

## Introduction

Hepatitis C virus (HCV) infects over 170 million people worldwide and is a major cause of chronic hepatitis, liver cirrhosis, and a high rate of hepatocellular carcinoma [1]. Chronic infection with HCV is often associated with extrahepatic manifestations (EHMs) such as mixed cryoglobulinemia, non-Hodgkin lymphoma, thyroiditis and diabetes mellitus [2]. A low level of HCV-RNA replication has been detected in both peripheral blood mononuclear cells (PBMCs) and neuronal tissues [3, 4], suggesting that EHMs might be induced by the extrahepatic propagation of HCV. If so, however, the detailed mechanisms remain unknown.

Although there is no effective vaccine against HCV due to its genetic variability (quasispecies), the recent development of direct-acting antivirals (DAA) targeting viral proteins, such as NS3-4A protease, NS5A or NS5B polymerase [5], has improved the outcome. However, it has been reported that during DAA treatment, adaptive mutations in the HCV genome can be readily induced due to the low fidelity of NS5B RNA polymerase [6], resulting in the emergence of drug-resistant viruses [7–9].

A liver-specific microRNA (miRNA), miR-122, has been shown to be one of the most important host factors for HCV replication [10]. In general, miRNA negatively regulates the translation of mRNA through an interaction with the 3’UTR in a sequence-specific manner. miR-122 regulates gene expressions important for functions involved in the maintenance of liver homeostasis, including the functions of lipid metabolism, iron metabolism, and carcinogenesis [11, 12]. On the other hand, the HCV 5’UTR has two binding sites for miR-122, which are highly conserved beyond the HCV genotype [13]. Upon HCV infection, miR-122 has been shown to stabilize HCV-RNA [14] and enhance internal ribosome entry site (IRES)-mediated translation [10, 15, 16] and replication [17] of HCV-RNA through direct interaction with the HCV 5’UTR [18, 19]. However, the detailed function of miR-122 in the HCV lifecycle has not been understood well. In addition, this interaction results in the sequestration of miR-122 from host mRNA targets, which may be responsible for the long-term oncogenic potential of HCV [20].

Recent studies have revealed the importance of Ago2, a catalytic component of the RNA-induced silencing complex (RISC), and of Xrn1 and Xrn2, 5’ to 3’exonucleases involved in mRNA decay, on the stabilization of HCV-RNA; however, the detailed mechanisms are largely unclear. Ago2 participates in the stabilization and enhancement of translation and replication of HCV-RNA via direct interaction with miR-122 and the HCV 5’UTR [21, 22], and Xrn1 and Xrn2 are involved in degradation of HCV-RNA [23–25], while miR-122 might protect the HCV genome from such degradation. A locked nucleic acid (LNA) complementary to miR-122 has advanced to phase II clinical trial, and subcutaneous injection of LNA into chronic hepatitis C patients was shown to suppress the propagation of HCV without the appearance of any drug-resistant virus or adaptive mutation in the 5’UTR of the HCV genome [26]. Recently, however, the G28A mutation adjacent to the miR-122 binding site in the 5’UTR of the genotype 2a Jc1 genome introduced during infection in the presence of an miR-122 decoy has been shown to possess more potent accessibility and affinity to miR-122 [27]. In addition, we and another group have previously shown that a low level of replication of HCV occurred in several miR-122-deficient cells including non-hepatic Hec1B and hepatoma Hep3B cells [28, 29], suggesting that HCV-RNA is capable of propagating in an miR-122-independent manner.

In this study, we investigated the roles of miR-122 not only on replication of HCV-RNA but also on production of infectious particles by using miR-122-knockout Huh7 cells. Adaptive HCV mutants obtained after serial passages in miR-122-knockout cells exhibited efficient RNA replication in an miR-122-independent manner by introduction of the G28A mutation for miR-122-independence and several mutations for higher infectivity. The emergence of an HCV mutant that can propagate in an miR-122-independent manner may participate in the induction of extrahepatic manifestations in chronic hepatitis C patients.

## Results

### Establishment of miR-122-knockout Huh7 cells

To clarify the biological significance of miR-122 in the life cycle of HCV in more detail, we established miR-122-knockout Huh7 (Huh7-122KO) cells by using artificial endonucleases (transcription activator-like effector nucleases: TALEN). Two clones (clone #1 and #2) of Huh7-122KO cells were obtained and mutations in the miR-122-coding region were assessed by Surveyor nuclease assay and sequencing analysis (S1A and S1B Fig). Lack of expression of miR-122 in Huh7-122KO cells was confirmed by Northern blotting and qRT-PCR (S1C Fig). In addition, we established Huh7-122KO cells in which the expression of miR-122 was recovered by infecting Huh7-122KO#1 and Huh7-122KO#2 cells with a lentiviral vector expressing miR-122 and obtained Huh7-122KOR#1 and Huh7-122KOR#2 cells, respectively. Next, to confirm that the loss of miR-122 activity suppressed translation of the target gene in Huh7-122KO cells, a pmirGLO vector carrying the complementary sequence of miR-122 under the luciferase gene was transfected into Huh7-122KO cells. Suppression of luciferase activity was observed not only in parental Huh7 cells, but also in Huh7-122KOR#1 and Huh7-122KOR#2 cells, while no suppression was observed in Huh7-122KO#1 and Huh7-122KO#2 cells (S1D Fig). Furthermore, we confirmed that cell growth and viability of Huh7-122KO cells were comparable to those of restored cells (S2 Fig). These results suggest that Huh7-122KO cells are functionally deficient in miR-122 activity and exogenous expression of miR-122 restored their biological function.

### miR-122 is required for replication complex formation in the early phase of infection but not for maintenance of replication

**Fig 1:**
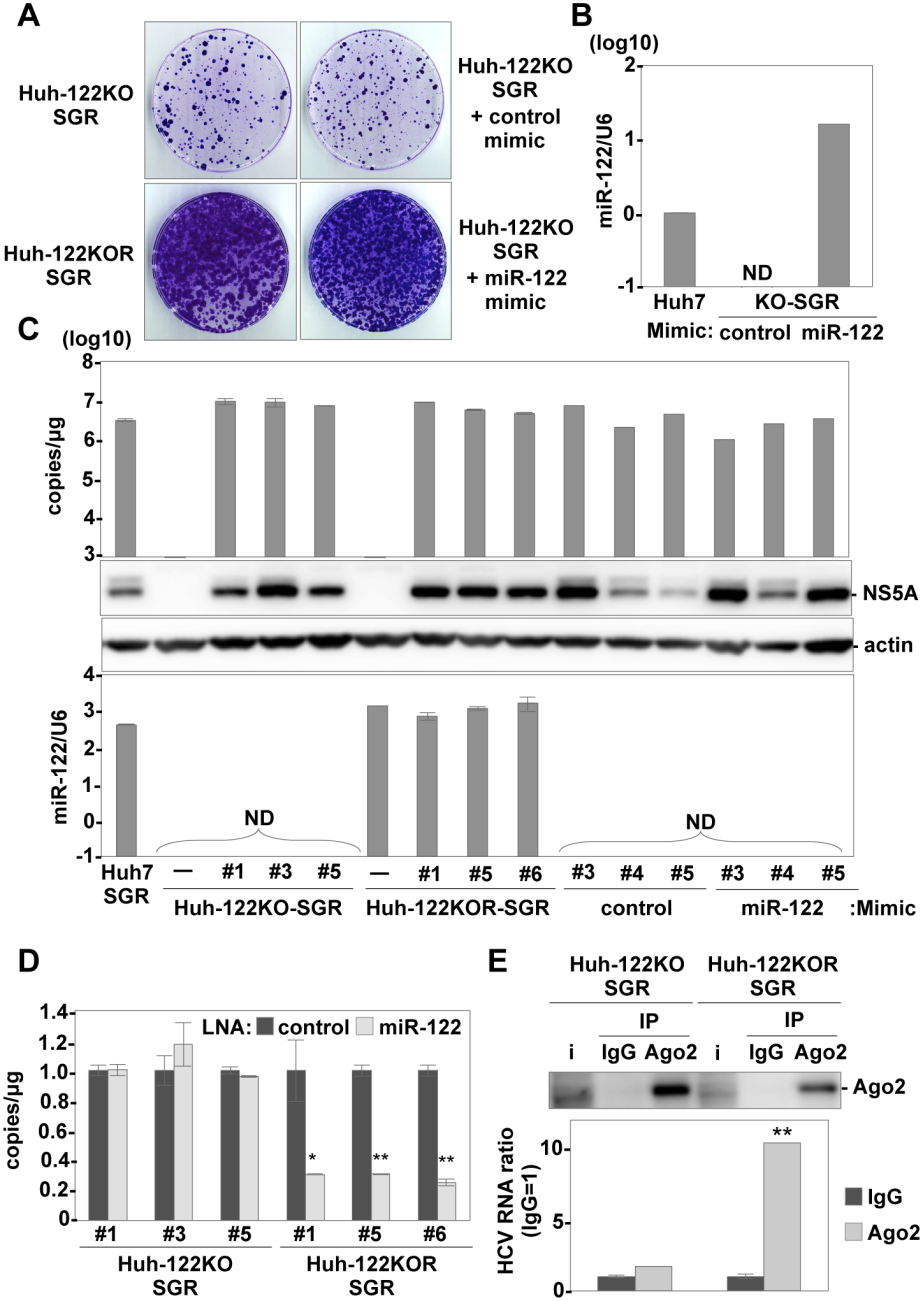
Establishment of replicon cells derived from Huh7-122KO cells. (A) Subgenomic HCV replicon RNA was electroporated into Huh7-122KO and Huh7-122KOR cells, or into Huh7-122KO cells together with control- or miR-122-mimic, and G418-resistant colonies were stained with crystal violet at 21 days post-transduction. (B) Expression of miR-122 in Huh7-122KO cells electroporated with either control-mimic or miR-122-mimic at 72 h post-electroporation. Relative expression of miR-122 was determined by qRT-PCR by using U6 snRNA as an internal control. (C) Each of the three clones derived from each type of replicon cells was subjected to qRT-PCR after extraction of total RNA (top) and to immunoblotting by using anti-NS5A and β-actin (middle). The relative expression of miR-122 was determined by qRT-PCR by using U6 snRNA as an internal control (bottom). (D) Intracellular HCV-RNA replication in Huh7-122KO-SGR cells (#1, #3, #5) and Huh7-122KOR-SGR cells (#1, #5, #6) in the presence of 20 nM of either LNA-control or LNA-miR122 was determined by qRT-PCR. (E) The Ago2 complex was immunoprecipitated from Huh7-122KO-SGR#1 and Huh7-122KOR-SGR#1 cells by using either anti-IgG or anti-Ago2 mouse antibody. The HCV-RNA associated with Ago2 was determined by qRT-PCR and Ago2 was detected by immunoblotting. Error bars indicate the standard deviation of the mean and asterisks indicate significant differences (*P < 0.05; **P < 0.01) versus the results for the control.

First, we compared the expression levels of host mRNAs between Huh7-122KO and Huh7-122KOR cells by cDNA microarray. From the results of pathway prediction in Huh7-122 KO cells by Ingenuity Pathway Analysis (IPA), the expression levels of genes involved in lipid metabolism were changed in Huh7-122KO cells (S1 Table), as in previous reports on miR-122-knockout mice [11, 12]. Previous reports have shown that lipid metabolism participates in the entry or assembly of HCV. Therefore, to examine the effect of miR-122-knockout-induced changes in the lipid composition on the entry of HCV, pseudotyped VSV bearing HCV envelope proteins, HCVpv, was inoculated into the cell lines. No difference in luciferase activity was observed irrespective of miR-122 expression upon infection with HCVpv (S3 Fig), suggesting that the knockout of miR-122 has no significant effect on the entry of HCV. Next, to investigate the function of miR-122 on the replication of viral RNA, we examined the effects of stable and transient expression of miR-122 in the miR-122-knockout cells on colony formation. HCV subgenomic replicon (SGR) RNA of the JFH1 strain was electroporated into Huh7-122KO and Huh7-122KOR cells. The numbers of colonies were significantly higher in Huh7-122KOR cells than in Huh7-122KO cells (Fig 1A, left panels), while the expression of NS5A and formation of membranous webs were similarly observed in both Huh7-122KO-SGR and Huh7-122KOR-SGR cells (S4A and S4B Fig), respectively. In addition, the inhibitory effect by treatment with IFNα and BILN was comparable with Huh7-122KO-SGR cells (S5 Fig). Although higher levels of miR-122 expression in Huh7-122KO cells transduced with the mature form of miR-122 (miR-122-mimic) were detected at 72 h post-electroporation (Fig 1B), no miR-122 was detected at the time of colony harvest (at 21 days post-electroporation) due to the instability of miR-122-mimic (Fig 1C, lower panel). However, enhancement of colony formation was observed upon electroporation of SGR RNA together with miR-122-mimic but not of control-mimic in Huh7-122KO cells (Fig 1A, right panels), suggesting that not continuous but transient expression of miR-122 is sufficient for the enhancement of colony formation. Next, we confirmed that HCV SGR RNA in miR-122 KO cells could replicate independently from miR-122. High levels of HCV-RNA replication were detected in Huh7-122KO-SGR cells (Fig 1C, upper panel), which were resistant to the treatment with miR-122 inhibitor, in contrast to Huh7-122KOR-SGR cells (Fig 1D). In addition, more abundant HCV-RNA was detected in the Ago2 complex immunoprecipitated with a specific antibody in Huh7-122KOR-SGR cells than in Huh7-122KO-SGR cells (Fig 1E). These results suggest that miR-122 is required in the early phase for the efficient translation of viral RNA crucial for formation of the replication complex, and transient expression of miR-122 is able to rescue viral replication in Huh7-122KO cells. However, once the replication complex is formed, the HCV genome can replicate in an miR-122-independent manner.

### miR-122-independent propagation of HCV

**Fig 2:**
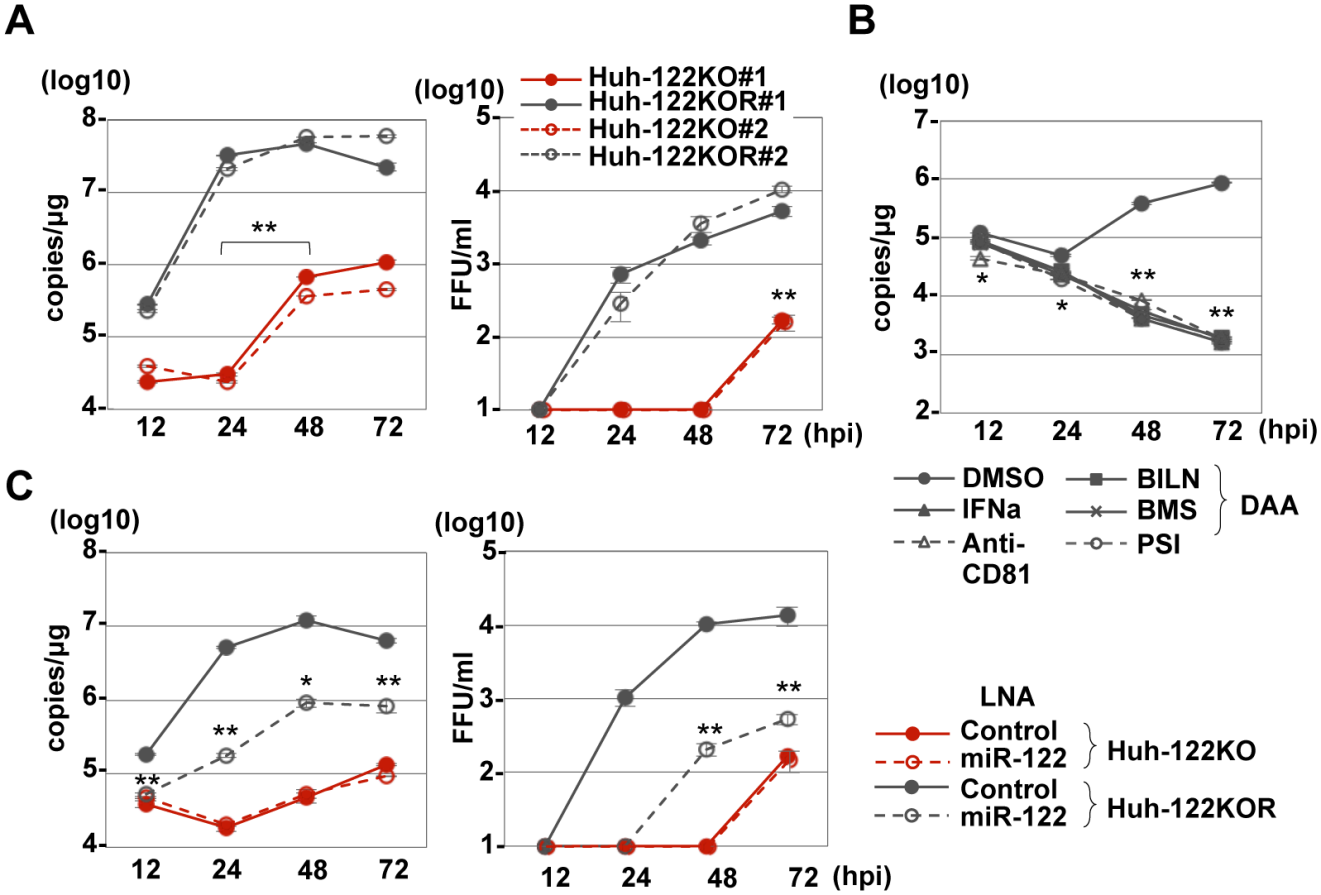
miR-122-independent HCV replication in Huh7-122KO cells. (A) HCV was inoculated into Huh7-122KO and Huh7-122KOR cells at an MOI of 3, and intracellular HCV-RNA levels (left panel) and infectious titers in the culture supernatants (right panel) were determined by qRT-PCR and focus formation assay, respectively. (B) HCV-RNA replication was inhibited by the treatment with IFNα, BILN, BMS790052, PSI7977 and anti-CD81 antibody. (C) HCV replication in Huh7-122KO cells was resistant to treatment with an miR-122 inhibitor, LNA (HCV-RNA replication: left; infectious titer: right). Error bars indicate the standard deviation of the mean and asterisks indicate significant differences (*P < 0.05; **P < 0.01) versus the results for the control.

Next, we examined the effect of the knockout of miR-122 in Huh7 cells on the propagation of HCV. Although Huh7-122KOR cells exhibited efficient HCV replication, a slight increase of intracellular HCV-RNA levels and detectable infectious titers in the culture supernatants was observed in Huh7-122KO cells upon infection with HCV at an MOI of 3 (Fig 2A). To rule out the possibility of incorporation of miR-122 into viral particles, *in vitro* transcribed full-genomic HCV-RNA was electroporated into Huh7-122KO cells together with either control- or miR-122-mimic, and infectious titers in the culture supernatants were determined (S6 Fig). A slight but significant increase of infectious titer was detected in the culture supernatants of Huh7-122KO cells co-electroporated with viral RNA and control-mimic. To further confirm the miR-122 independent propagation of HCV, we examined the effect of inhibitors for HCV, including IFNα, DAA and anti-CD81 antibody, on the propagation of HCV in Huh7-122KO cells. The level of HCV-RNA in Huh7-122KO cells upon infection with HCV was decreased by the treatment with the reagents (Fig 2B). However, as we expected, the treatment with LNA-miR-122 exhibited no effect on the replication of HCV in Huh7-122KO (Fig 2C). Previous reports showed that a specific membrane alteration that is involved in viral replication, i.e., the formation of a membranous web, was observed in HCV-replicating cells [30]. Correlative fluorescence microscopy-electron microscopy (FM-EM) also revealed the localization of NS5A on convoluted structures such as the membranous web in Huh7-122KO cells (S7 Fig). Moreover, HCV core proteins and lipid droplets were co-localized in Huh7-122KO cells (S8 Fig), suggesting that HCV can propagate in an miR-122-independent manner.

**Fig 3:**
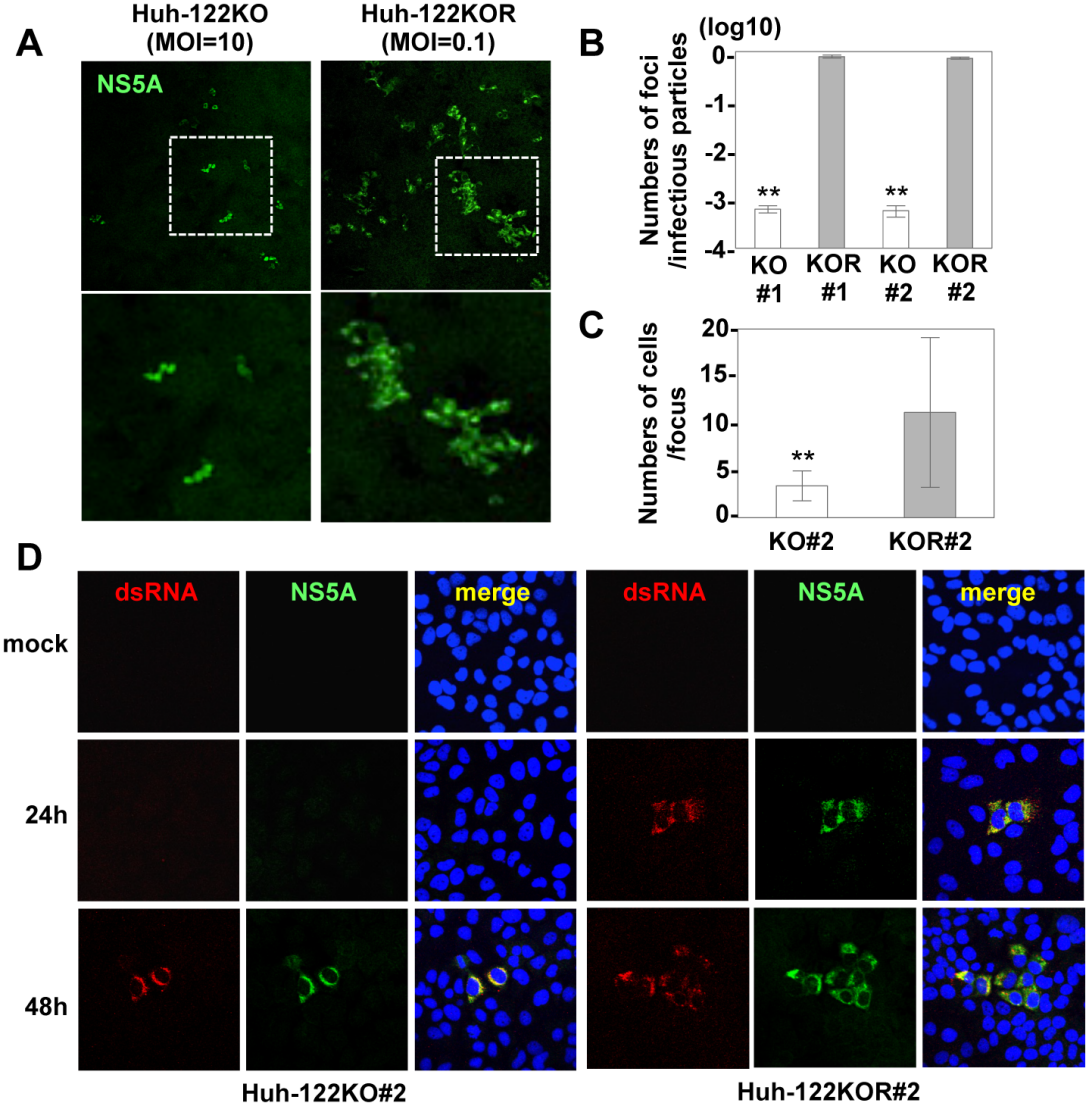
Focus formation in Huh7-122KO cells. (A) Huh7-122KO (left) and Huh7-122KOR cells (right) infected with HCV at an MOI of 10 or 0.1, respectively, were fixed at 72 hpi and stained with antibodies to NS5A protein (green). The boxes in the top panels were magnified (bottom panel). Numbers of foci per infectious particle (B) and numbers of cells per focus (C, average of 75 foci) in Huh7-122KO (white bar) and Huh7-122KOR cells (gray bar) upon infection with HCV are shown. Error bars indicate the standard deviation of the mean and asterisks indicate significant differences (**P < 0.01) versus the results for the control. (D) Focus formation in Huh7-122KO (left) and Huh7-122KOR cells (right) infected with HCV at an MOI of 0.1 or 10, respectively. Each cell was fixed at the indicated time post-infection and stained with appropriate antibodies to dsRNA (red) and NS5A (green). Cell nuclei were stained with DAPI (blue).

Next, to examine the role of miR-122 on the spread of HCV infection, we compared the focus formation in Huh7-122KO and Huh7-122KOR cells at 72 h post-infection. The size and number of foci formed in Huh7-122KO cells were smaller and fewer than those in Huh7-122KOR cells (Fig 3A). Focus-formation efficiency was 1000-fold lower in Huh7-122KO cells than in Huh7-122KOR cells upon infection with HCV at an MOI of 1 (Fig 3B). In addition, the average numbers of infected cells constituting one focus in Huh7-122KO cells (approximately 3 cells/focus) were significantly smaller than those in Huh7-122KOR cells (approximately 10 cells/focus) (Fig 3C). To investigate the propagation of HCV in Huh7-122KO cells in more detail, the expression of dsRNA and NS5A protein in cells upon infection with HCV was assessed by immunofluorescence analysis. Co-localization of dsRNA and NS5A in Huh7-122KOR cells was observed at 24 h post-infection, but was detected at 48 h post-infection in Huh7-122KO cells (Fig 3D), supporting the hypothesis that miR-122 is required for the efficient formation of the replication complex as shown by replicon assay. Because expression of lipoprotein-associated ApoB, ApoE or MTTP was decreased in Huh7-122KO cells (S9 Fig), we examined the involvement of miR-122 in HCV particle formation. However, no significant difference was observed in particle formation relative to intracellular copy number at 72 h post-infection between Huh7-122KO and Huh7-122KOR cells (S10 Fig), suggesting that miR-122 does not participate in infectious particle formation. Collectively, these results suggest that the low levels of focus formation of HCV in Huh7-122KO cells are attributable to inefficient translation and replication of the HCV genome, but not to the particle formation process.

### miR-122-knockout Huh7.5.1 cells exhibit a high susceptibility to HCV infection

Generally, it has been reported that cured cells established by the elimination of viral RNA from HCV replicon cells exhibited more potent propagation of HCV than parental cells. Previous reports have shown that the high susceptibility of cured cells to HCV infection is due to the high level of miR-122 expression [31–33]. On the other hand, Huh7.5.1 cells, which are also derived from HCV replicon cells, carry mutations in RIG-I, a key innate immune sensor for viral RNA [34] and express a low level of CREB3L1/OASIS, which specifically inhibits the proliferation of cells infected with HCV [35].

**Fig 4:**
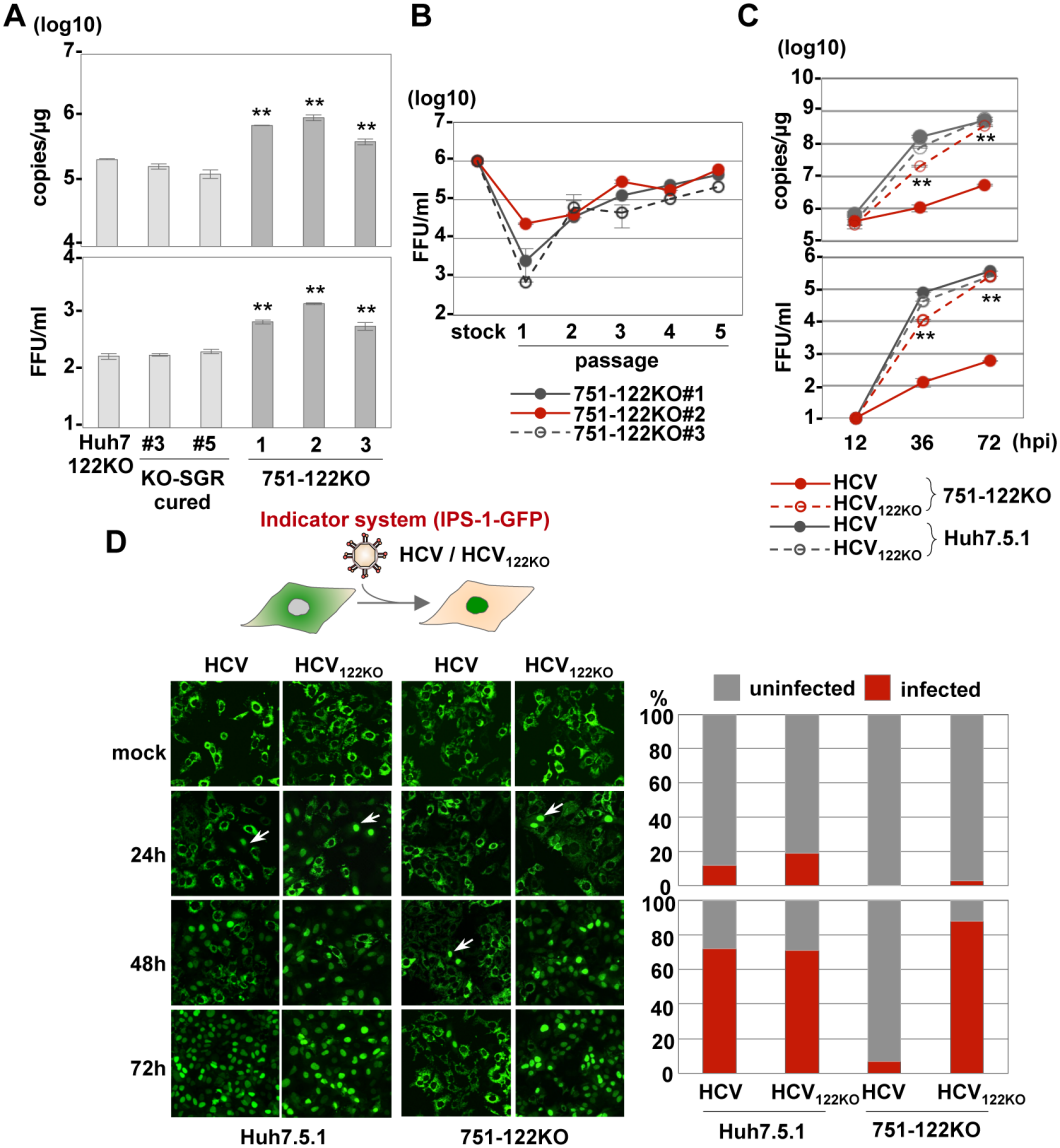
Propagation of HCV_122KO_ in 751-122KO cells. (A) HCV was inoculated into Huh7-122KO (#2), Huh7-122KO-cured (#3 or #5), or 751-122KO (#1, #2 or #3) cells, and the levels of intracellular HCV-RNA replication (top) and infectious titers in the culture supernatants (bottom) were determined by qRT-PCR and focus formation assay, respectively, at 72 hpi. (B) Infectious titer in the culture medium on serial passage of each 751-122KO cell clone. (C) HCV and HCV_122KO_ were inoculated into 751-122KO and Huh7.5.1 cells and the levels of intracellular HCV-RNA replication (top) and infectious titers in the culture supernatants (bottom) were determined at 72 hpi. Error bars indicate the standard deviation of the mean and asterisks indicate significant differences (*P < 0.05; **P < 0.01) versus the results for the control. (D) Nuclear translocation of IPS-GFP (arrows) in Huh7.5.1 and 751-122KO cells upon infection with HCV and HCV_122KO_ (left panels). The numbers of cells having translocated GFP in their nuclei through propagation of HCV were counted and the infection ratios at 24 hpi (right top) and 72 hpi (right bottom) were determined.

To determine the difference in propagation of HCV in the absence of miR-122 between Huh7 and Huh7.5.1 cells, we established 3 clones of miR-122-knockout Huh7.5.1 (751-122KO) cells (#1- #3) and the miR-122 deficiency in each clone was confirmed by sequencing analysis and qRT-PCR (S11A and S11B Fig). The results showed that RNA replication and infectious particle formation in the supernatants of 751-122KO cells were higher than those in Huh7-122KO cells or cured cells upon infection with HCV at an MOI of 1 (Fig 4A). To further confirm the difference of infectivity between parental and cured cells, we also established cured cells from Huh7-122KO-SGR cells (#3 and #5) by treatment with IFNα and BILN (S12 Fig). However, the infectivity of cured cells was comparable to that of parental Huh7-122KO cells, suggesting that the high susceptibility of Huh7.5.1 cells to HCV infection depends not only on a high level expression of miR-122 but also on other factors.

### An adapted mutant, HCV_122KO_, can propagate in an miR-122-independent manner

Because 751-122KO cells exhibit higher susceptibility to HCV propagation than Huh7-122KO cells, we tried to generate an adapted mutant that could propagate in an miR-122-independent manner in 751-122KO cells. Infectious titers in the supernatants of 751-122KO cells upon infection with high titers of HCV were increased by cell passaging and reached 10^6^ FFU/ml (Fig 4B). The adapted HCV mutant capable of propagating in miR-122-knockout cells was designated HCV_122KO_. Although HCV propagated well in Huh7.5.1 cells, the intracellular HCV-RNA levels and infectious titers in the supernatants were impaired in 751-122KO cells. In contrast, HCV_122KO_ exhibited an efficient and comparable propagation in both cell lines irrespective of the expression of miR-122 (Fig 4C). Next, to confirm miR-122-independent propagation of HCV_122KO_, replication of HCV was assessed by an indicator system [36], which monitored HCV propagation by the nuclear localization of the IPS-1-GFP fusion protein through cleavage by NS3-4A protease upon infection with HCV. Although nuclear localization of GFP was similarly detected from 24 h post-infection and completed at 72 h post-infection in Huh7.5.1 cells infected with either HCV or HCV_122KO_, it was severely impaired in 751-122KO cells infected with HCV, and only small numbers of GFP molecules were detected in the nucleus at 48 h post-infection. In contrast, nuclear localization of GFP comparable to that in Huh7.5.1 cells was observed in 751-122KO cells upon infection with HCV_122KO_ (Fig 4D), suggesting that HCV_122KO_ is capable of propagating in an miR-122-independent manner.

### Efficient propagation of HCV_122KO_ in several miR-122-deficient cell lines

**Fig 5:**
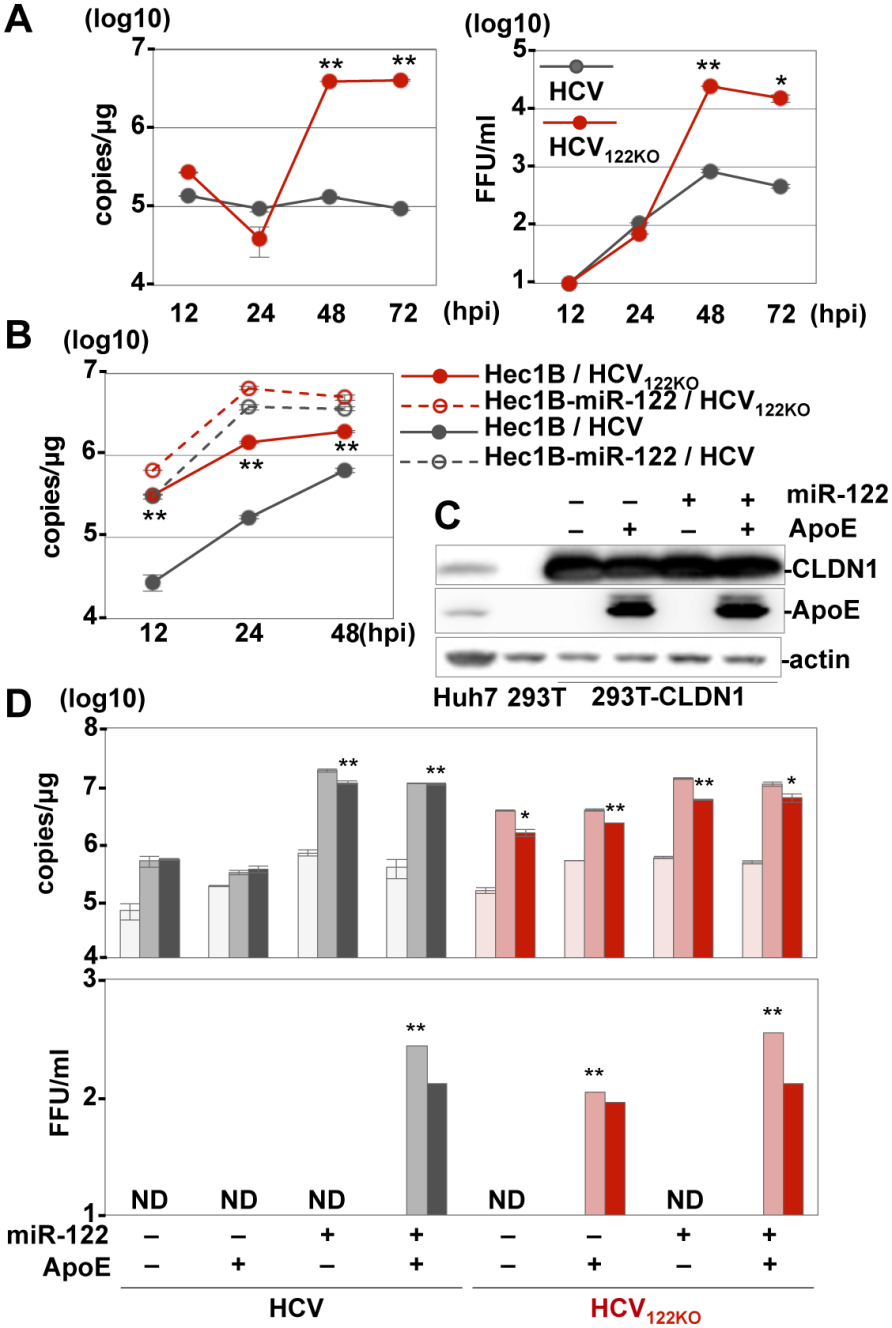
miR-122-independent propagation of HCV_122KO_. (A) Intracellular HCV-RNA levels (left panel) and infectious titers in the culture supernatants (right panel) of Hep3B cells infected with HCV or HCV_122KO_ were determined. (B) Hec1B cells with or without exogenous expression of miR-122 were infected with HCV or HCV_122KO_ and the levels of intracellular HCV-RNA were determined. (C) Immunoblotting of 293T-CLDN cells with exogenous expression of miR-122 and ApoE. (D) 293T-CLDN cells were infected with either HCV or HCV_122KO_ and the levels of intracellular HCV-RNA (upper) and infectious titers in the culture supernatants (lower) were determined at 12, 36 and 72 hpi (horizontal). Error bars indicate the standard deviation of the mean and asterisks indicate significant differences (*P < 0.05; **P < 0.01) versus each result at 12 hpi.

To verify the ability of HCV_122KO_ to propagate efficiently in an miR-122 independent manner, several miR-122-deficient cells, including non-hepatic cells, were infected with HCV_122KO_. miR-122-independent intracellular RNA replication and infectious particle formation of HCV_122KO_ was observed in Hep3B cells deficient in miR-122 expression [29] (Fig 5A). Moreover, compared to HCV, HCV_122KO_ exhibited more potent replication comparable to that by the overexpression of miR-122, as previously reported [28] in uterus-derived Hec1B cells (Fig 5B) and other non-hepatic cell lines, including A549 (lung), MC-IXC (neuron), Caki-2 (kidney) and RERF-LC-AI (lung) (S13 Fig) and primary HMSC with overexpression of CLDN1 and OCLN (S14 Fig). However, no infectious particles were detected in the culture supernatants of the non-hepatic cell lines infected with HCV_122KO_. It has been reported that ApoE plays crucial roles in the efficient production of infectious particles in 293T cells expressing Claudin1 (293T-CLDN1) [37]. Although co-expression of miR-122 and ApoE is required for the production of infectious particles in 293T-CLDN1 cells upon infection with HCV, expression of ApoE alone permits particle formation in cells infected with HCV_122KO_ (Fig 5C and 5D). In addition, the replications of HCV and HCV_122KO_ in non-hepatic Hec1B and 293T-CLDN1 cells were also inhibited by the NS3-4A inhibitor BILN as observed in Huh7-122KO cells (S15 Fig), suggesting that HCV_122KO_ can propagate in non-hepatic cells in the absence of miR-122.

### Identification of mutations in the 5’UTR of HCV_122KO_

**Fig 6:**
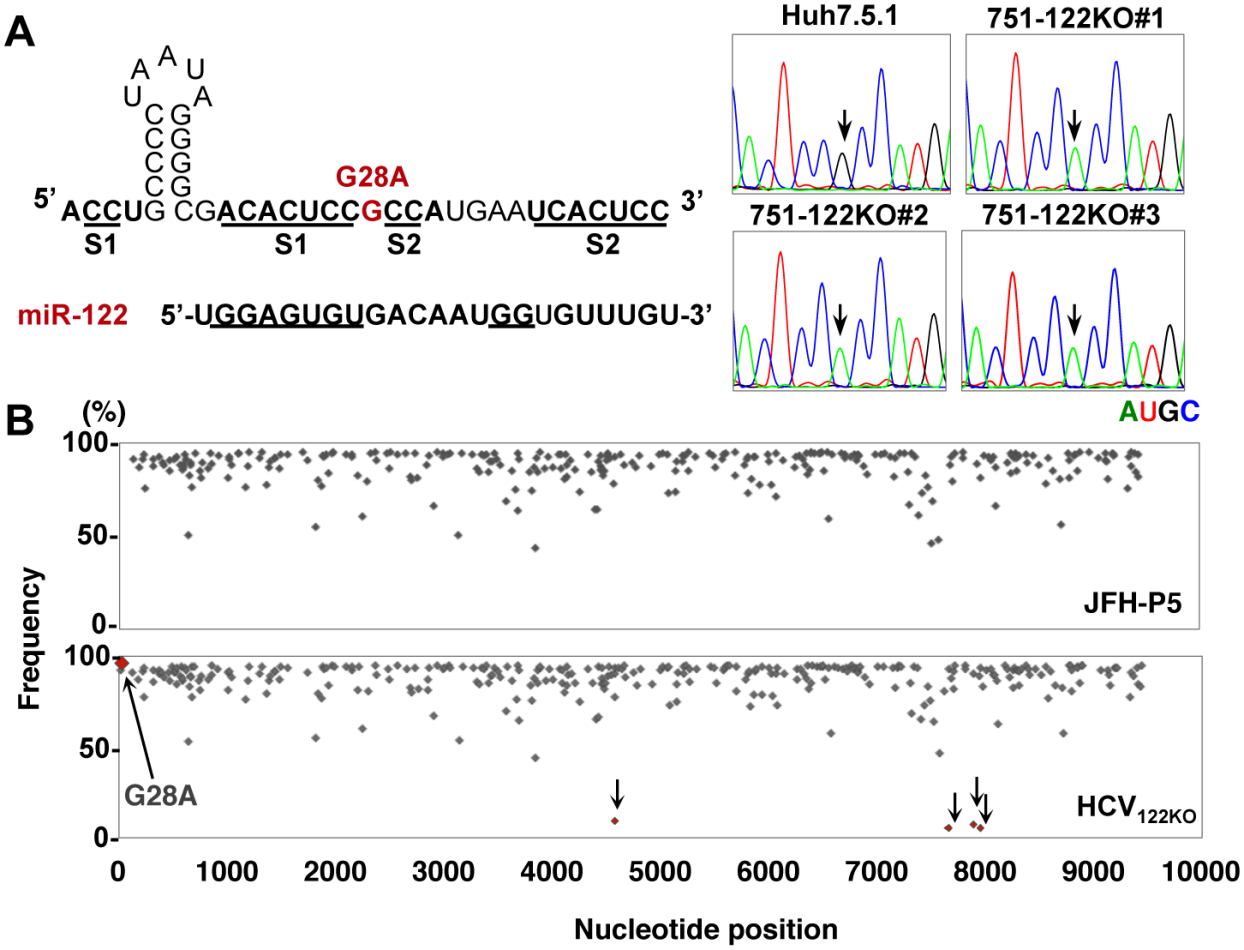
Identification of adaptive mutation in HCV_122KO_. (A) Mutation of G28A in the 5’UTR of HCV was identified in all independently isolated HCV propagated in the three 751-122KO cell clones (751-122KO#1~#3). Arrows indicate the position of nt28 in the 5’UTR of HCV. Each RNA base is represented as a colored peak: A, green; U, red; G, black; and C, blue. (B) Frequency and distribution of SNV in HCV independently cultured in Huh7.5.1 (JFH-P5; top) and 751-122KO cell clones (bottom). Six independently isolated HCV_122KO_ viruses were obtained from three wells for each of two 751-122KO cell clones (751-122KO#1 and #2). Each sequence read was mapped to pHH-JFH1-E2p7NS2mt. Arrows indicate the detected substitutions.

To clarify the molecular mechanisms of the miR-122-independent propagation of HCV_122KO_, we first confirmed the introduction of adaptive mutations into the viral genome of three independently isolated HCV_122KO_ by direct sequencing analyses. The mutation of G28A in the 5’UTR of HCV was identified in all independently isolated HCV propagated in 751-122KO cell clones (751-122KO-#1~#3) (Fig 6A). To analyze this result in more detail, deep sequencing analysis of a mixture of six independently isolated HCV_122KO_ was performed. Deep sequencing by Pac-bio enables long-reads (~3 kbp) and detects single nucleotide variants (SNVs) including accidental sequencing errors. Compared with HCV at five serial passages in Huh7.5.1 cells (JFH-P5), only the dominant mutation G28A was observed from a mixture of six independently isolated HCV_122KO_ (Fig 6B and S16A–S16C Fig). We also identified a few synonymous substitutions at nucleotide position (nt) 4579 in NS3, nt7658 in NS5A and nt7888 or nt7951 in NS5B at a low rate; however, they were from different fragments of cDNA of HCV-RNA. These results indicated that, except in the case of G28A, these mutations arise irregularly, and are not specific to an miR-122-deficient condition. In addition, G28A mutation also emerged by the transfection of a plasmid encoding a full-genomic HCV cDNA, pHH-JFH1-E2p7NS2mt, into 751-122KO cells (S17A and S17B Fig), while there was no adaptive mutation in HCV SGR RNA from replicon cells (S17C Fig). Therefore, we examined the difference between the G28A virus and HCV_122KO_ infectivity in Huh7-122KO and Huh7-122KOR cells (S18 Fig). Compared to the wild type HCV, both the G28A and HCV_122KO_ virus showed miR-122-independence, while HCV_122KO_ had higher infectivity than the G28A virus both in Huh7-122KO and Huh7-122KOR cells. Therefore, we concluded that not only the G28A mutation responsible for miR-122-independence but also other adaptive mutations involved in higher replicative fitness are required for efficient propagation in an miR-122-independent manner.

### An adapted virus from gt1b/2a chimeric HCV also propagates in miR-122-deficient cells in an miR-122-independent manner

**Fig 7:**
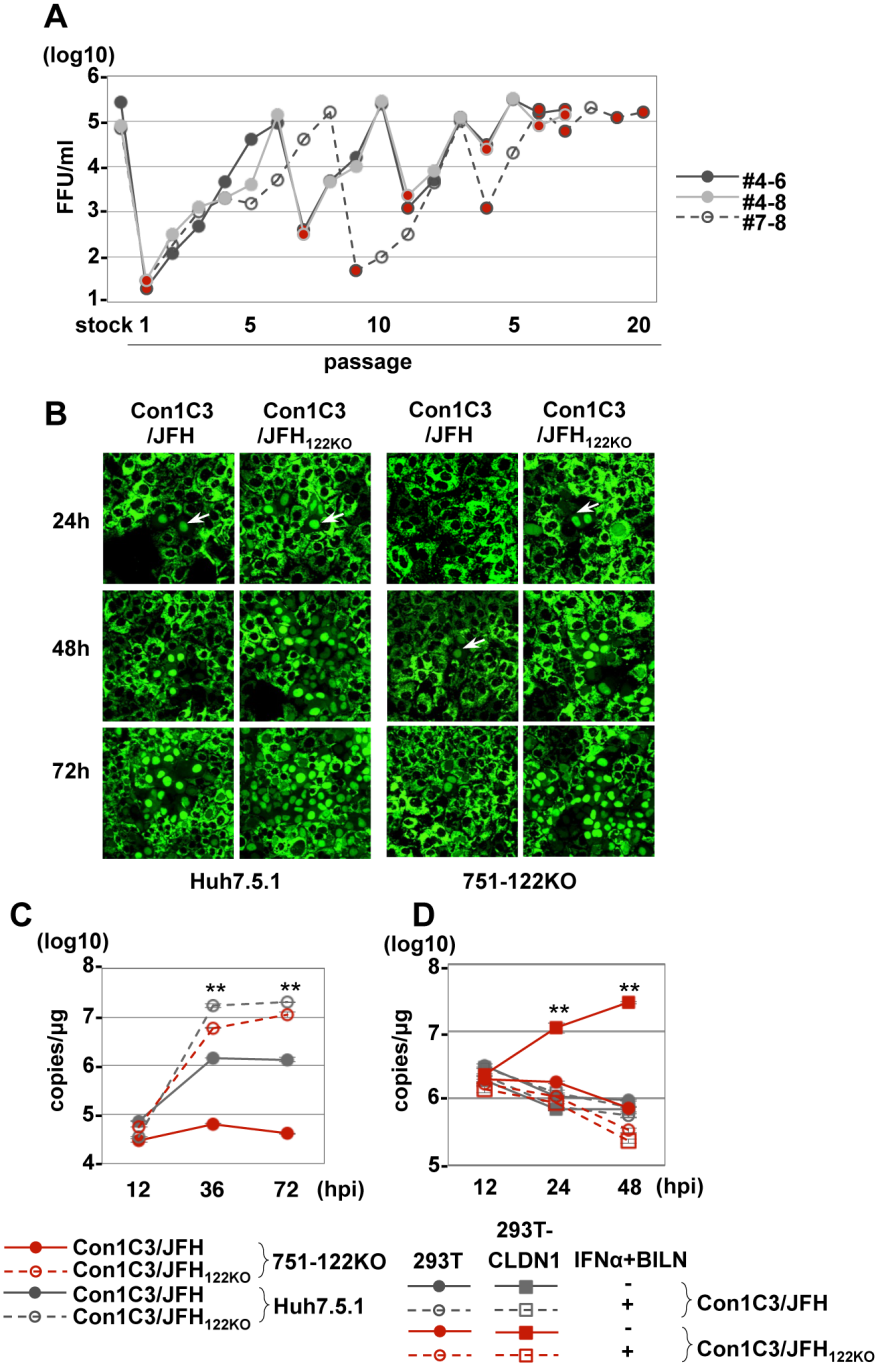
Propagation of Con1C3/JFH_122KO_ in 751-122KO cells. (A) Infectious titer in the culture medium on serial passage of 751-122KO#1 or Huh7.5.1 cells. Red circles indicate the passage in 751-122KO cells, and the other circles indicate the passage in Huh7.5.1 cells. Three independent passages (#4–6, #4–8, #7–8) are shown. (B) Nuclear translocation of IPS-GFP (arrows) in Huh7.5.1 and 751-122KO cells upon infection with Con1C3/JFH and Con1C3/JFH_122KO_. (C) Con1C3/JFH and Con1C3/JFH_122KO_ were inoculated into 751-122KO#1 and Huh7.5.1 cells, and the levels of intracellular HCV-RNA replication were determined. Error bars indicate the standard deviation of the mean and asterisks indicate significant differences (**P < 0.01) versus the results for the control. (D) 293T-CLDN cells infected with either Con1C3/JFH or Con1C3/JFH_122KO_ were treated with IFNα and BILN and then the intracellular HCV-RNA level was determined at 12, 24 and 48 hpi. Error bars indicate the standard deviation of the mean and asterisks indicate significant differences (**P < 0.01) versus the results for the control.

Because genotype 1b originally has adenine at nt29, which corresponds to the position of G28A in genotype 2, we tried to identify adaptive mutations that could facilitate efficient propagation in an miR-122-independent manner by using the chimeric virus of genotype 1b Con1 and 2a JFH1 strain (Con1C3/JFH) at the C3 loop in NS2, which includes the 5’UTR of Con1. Because serial passages of Con1C3/JFH only in 751-122KO cells failed due to the low efficiency of viral replication, both Huh7.5.1 and 751-122KO cells were used to obtain the adaptive virus. Infectious titers in the supernatants of 751-122KO cells were gradually increased during alternative passages and reached 10^5^ FFU/ml, and the resulting virus was designated Con1C3/JFH_122KO_ (Fig 7A). In addition to HCV_122KO_, Con1C3/JFH_122KO_ showed efficient propagation both in Huh7.5.1 and 751-122KO cells (Fig 7B), while the HCV-RNA replication level of Con1C3/JFH_122KO_ was significantly higher than that of Con1C3/JFH both in Huh7.5.1 cells (Fig 7C) and in non-hepatic 293T-CLDN1 cells (Fig 7D). Therefore, we compared Con1C3/JFH and Con1C3/JFH_122KO_ by sequence analysis. Fifteen adaptive mutations were introduced throughout Con1C3/JFH RNA during serial passages (S19A Fig). Especially, G1A in 5’UTR has known to emerge during passages of gt1b replicon [38] or in gt1b patients [39]. Compared with Con1C3/JFH, Con1C3/JFH_122KO_ has additional 3 non-synonymous mutations located at the structural protein-coding region, 3 synonymous mutations at the non-structural protein-coding region and a single nucleotide mutation C30U in 5’UTR (S19B and S19C Fig). These results suggest that Con1C3/JFH_122KO_ also requires adaptive mutation that was directly involved in the role of miR-122 as with G28A mutation in 5’-UTR of JFH1 RNA to acquire miR-122-independence.

### Mutation of G28A in the 5’UTR of HCV_122KO_ facilitates miR-122-independent replication

**Fig 8:**
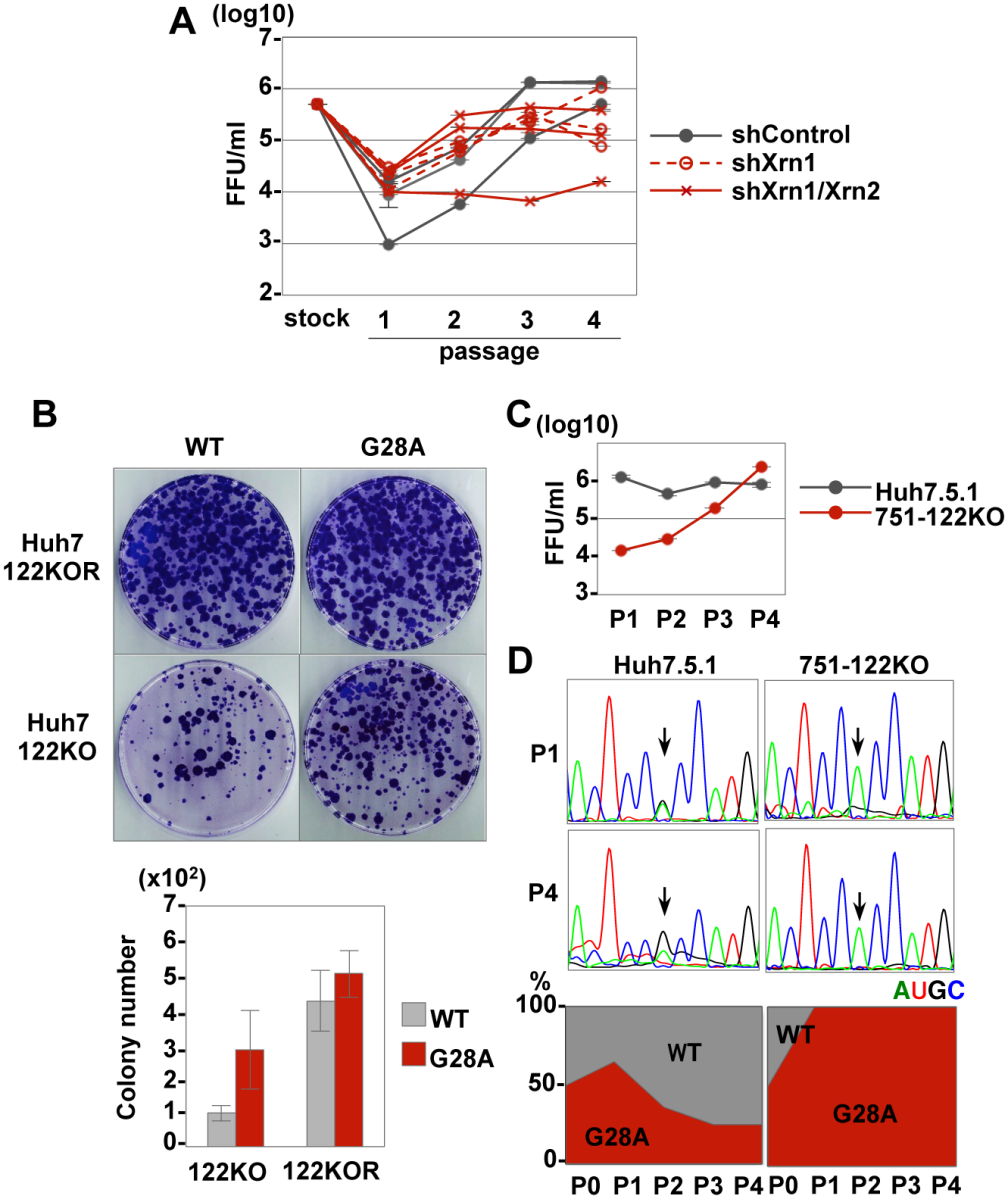
Effects of G28A mutation in the 5’UTR on the propagation of HCV. (A) Infectious titers in the culture media upon serial passage of three clones each of 751-122KO-shlacZ, 751-122KO-shXrn1 or 751-122KO-shXrn1/Xrn2 cells (#1~#3). (B) Colony formation in Huh7-122KO and Huh7-122KOR cells upon electroporation with the wild type and G28A-mutated JFH-SGR RNA (upper). The numbers of colonies of each cell type were quantified (bottom). Culture supernatants of 751-122KO and Huh7.5.1 cells co-electroporated with the wild type and G28A-mutated JFH1 HCV-RNA were harvested at each passage, and the infectious titers (C) and the sequences of viral RNA (D) were determined. Each RNA base is represented as a colored peak: A, green; U, red; G, black; and C, blue. Variations in the wild type and G28A mutant at passages 1 and 4 are shown (D, bottom). Error bars indicate the standard deviation of the mean and asterisks indicate significant differences (*P < 0.05; **P < 0.01) versus the results for the control.

Previous reports suggest that miR-122 binds to HCV 5’UTR and protects HCV-RNA from degradation by exonucleases Xrn1 or Xrn2 [23, 24] and Xrn1 plays a dominant role in this process [25]. To determine the roles of exonucleases in the introduction of G28A mutation under an miR-122-deficient condition, we generated three clones each of Xrn1 or both Xrn1 and Xrn2 stable knockdown cells based on miR-122 knockout Huh7.5.1 cells (751-122KO-shXrn1 or -shXrn1/Xrn2 cells) (S20A Fig). Infectious titers in the supernatants not only of 751-122KO control cells but also of 751-122KO-shXrn1 and 751-122KO-shXrn1/Xrn2 cells were gradually increased during serial passages (Fig 8A) with the emergence of G28A mutation (S20B Fig), suggesting that the emergence of G28A mutation in miR-122-deficient cells is independent of RNA decay by exonucleases.

Next, to clarify the effect of mutation of G28A in the 5’UTR of HCV_122KO_ on the replication of HCV-RNA, wild type or G28A-mutated JFH-SGR RNA was electroporated into Huh7-122KO and Huh7-122KOR cells. Although colony formation in Huh7-122KO cells transduced with G28A-mutated JFH-SGR RNA was higher than that in the cells transduced with the wild type RNA, there was no significant difference in colony formation between the Huh7-122KOR cells transduced with G28A and those transduced with wild type RNA (Fig 8B). Collectively, these results indicated that G28A mutation conferred a viral growth advantage in the absence of miR-122 by enhancing formation of the replication complex.

To examine the effect of G28A mutation on the propagation of HCV, wild type and G28A-mutated JFH1 RNAs were co-electroporated into Huh7.5.1 and 751-122KO cells (Fig 8C). Although infectious titers in the supernatants of Huh7.5.1 cells did not change appreciably with the number of passages, those of 751-122KO were increased by cell passaging. The G28A mutant exhibited higher replicative fitness for survival in 751-122KO cells, while replication of the G28A mutant was comparable to that of the wild type in Huh7.5.1 cells (Fig 8D). In addition, no revertant virus emerged during the eight serial passages of HCV_122KO_ in Huh7.5.1 cells (S21 Fig). These results suggest that the G28A mutant has a major advantage for propagation under an miR-122-deficient condition, but comparable infectivity to the wild type under an miR-122-rich condition.

### Association of G28A mutation with extrahepatic replication and manifestation

**Fig 9:**
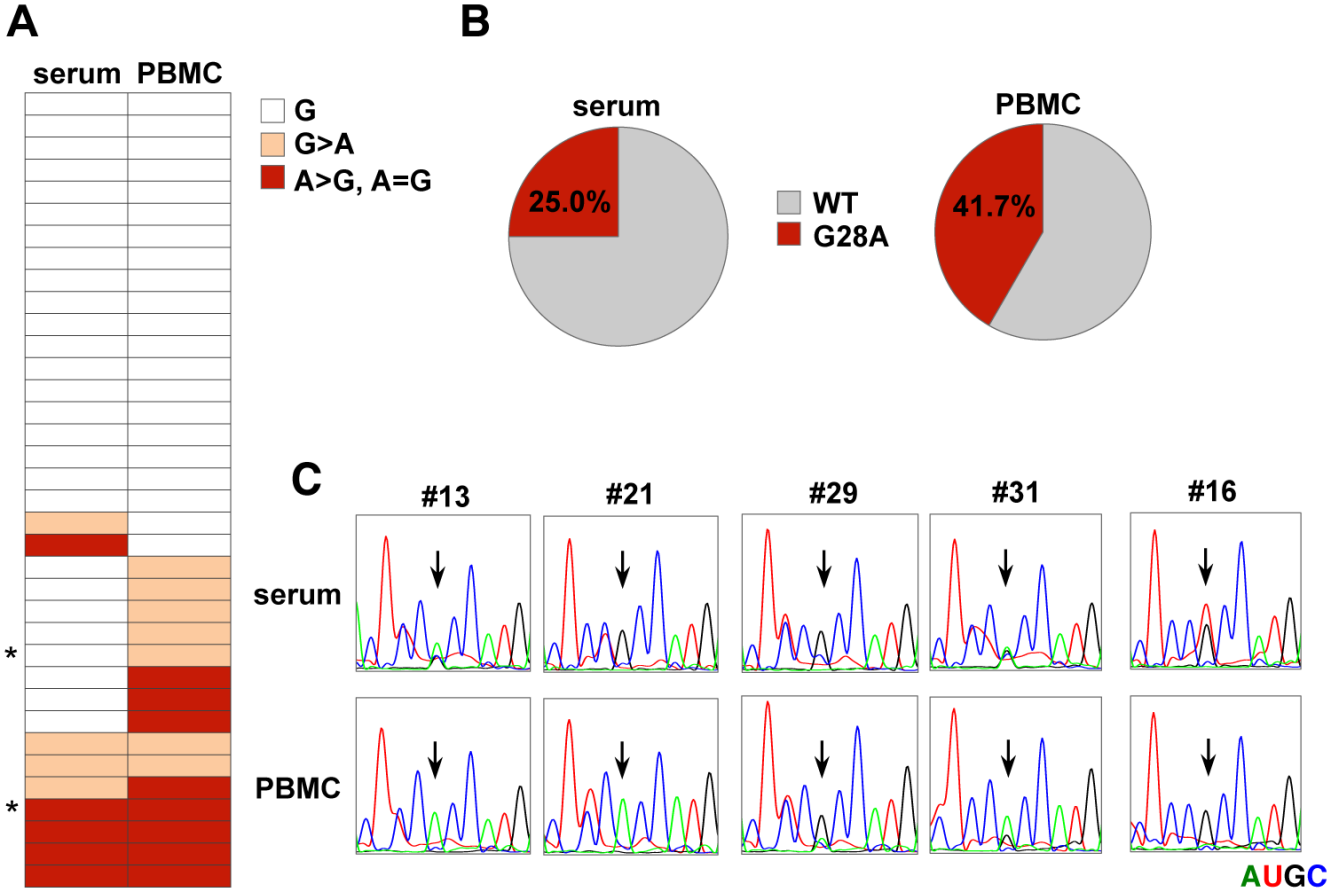
Detection of G28A mutation in HCV-RNA from the serum or PBMCs of gt2 patients. (A) Characterization of the nucleotide at nt28 from the serum and PBMCs of HCV gt2a patients. An asterisk indicates the samples from patients whose cases were complicated with hypothyroidism. (B) The ratio of samples between the WT and G28A from serum (left) or PBMCs (right). (C) Direct sequencing analysis. Viral RNA was purified from each PBMC or serum sample and subjected to sequencing analysis. Each RNA base is represented as a colored peak: A, green; U, red; G, black; and C, blue. Samples that included G28A (#13. #21, #29, #31) or G28U (#16) in either PBMCs or serum are shown.

Several studies have reported that HCV-RNA replication is detected in PBMCs, and malignant lymphoma sometimes occurs in chronic hepatitis C patients [3, 40–42], indicating the presence of persistent infection in the non-hepatic tissues of patients. Therefore, to examine whether or not G28A mutation is introduced into HCV-RNA replicating in non-hepatic cells *in vivo*, we analyzed the 5’UTR sequence of the HCV-RNA in PBMCs and hepatocyte-derived virus in the serum from genotype 2a patients (n = 36). Among these samples, 15 from PBMCs and 9 from serum showed emergence of the G28A mutation; notably, 7 patients had the G28A mutation in both PBMCs and serum (Fig 9A–9C), suggesting that the G28A mutation is induced dominantly in PBMCs and replication of the G28A virus is also maintained in hepatocytes, as shown by the competition assay (Fig 8C). We also found mutations from G to T (1 from serum) at nt28, though there were no mutations in the other regions. There were some patients with EHMs such as hypothyroidism (n = 2; #29 and #31 in Fig 9C) and malignant lymphoma (n = 1; #16 in Fig 9C). Interestingly, all of them had mutations at G28; the former two patients had the G28A and the latter patient the G28U mutation.

### Interaction of the G28A mutant with Ago2 is dispensable for HCV RNA replication in miR-122 KO cells

**Fig 10:**
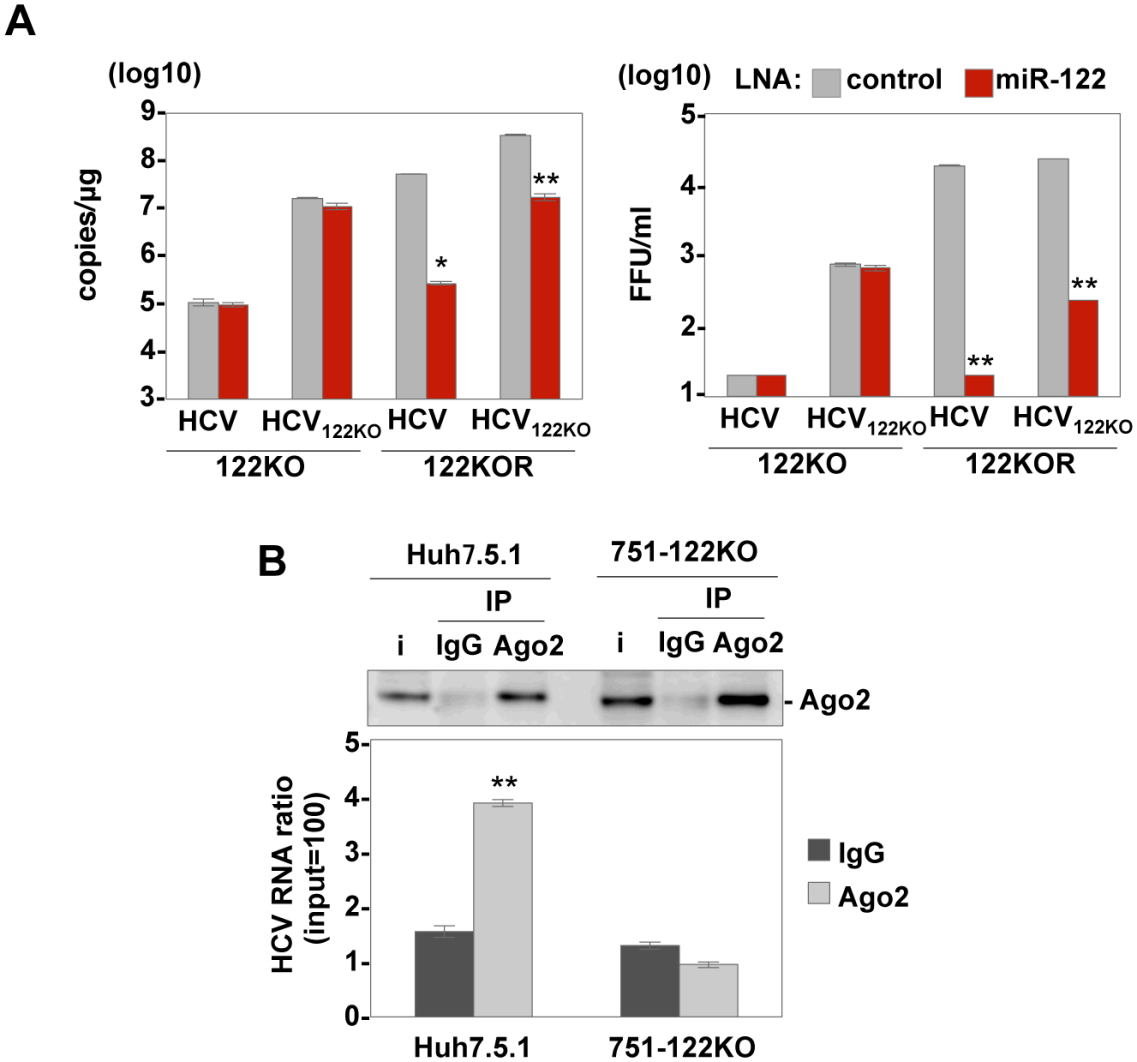
G28A mutants can replicate efficiently in an Ago2-independent manner. (A) Intracellular HCV-RNA levels (left panel) and infectious titers in the culture supernatants (right panel) of Huh7-122KO and Huh7-122KOR cells infected with either HCV or HCV_122KO_ in the presence of either control-LNA or LNA-miR-122 were determined at 72 hpi. (B) Ago2 complexes in 751-122KO and Huh7.5.1 cells infected with HCV were immunoprecipitated by either anti-IgG or anti-Ago2 mouse antibody at 12 dpi. Levels of Ago2 and HCV-RNA in the precipitates were determined by immunoblotting and qRT-PCR, respectively. Error bars indicate the standard deviation of the mean and asterisks indicate significant differences (*P < 0.05; **P < 0.01) versus the results for the control.

Because the HCV_122KO_ genome still contains two miR-122-binding sites in the 5’UTR, we further examined the effect of the miR-122 inhibitor on the propagation of HCV_122KO_ in Huh7-122KO and Huh7-122KOR cells (Fig 10A). Although intracellular viral RNA replication and infectious particle formation in the culture supernatants of Huh7-122KOR cells treated with the LNA control upon infection with HCV and HCV_122KO_ are comparable, those treated with LNA-miR-122 exhibited more potent suppression upon infection with HCV than HCV_122KO_. Interestingly, propagation of HCV_122KO_ in Huh7-122KOR cells was still significantly suppressed by the treatment with LNA-miR-122, suggesting that HCV_122KO_ utilizes miR-122 for its propagation, even in the presence of an abundance of miR-122. Next, to examine the effect of G28A mutation on the interaction with other miRNAs, Ago2 complexes in HCV infected Huh7.5.1 and 751-122KO cells were immunoprecipitated with anti-Ago2 antibody and the levels of Ago2 and HCV-RNA were determined by immunoblotting and qRT-PCR, respectively at 12 dpi. The interaction of Ago2 with viral genome was detected in Huh7.5.1 cells infected with HCV, but not in 751-122KO cells (Fig 10B), suggesting that no other miRNA helps to compensate for the role of miR-122 on HCV replication in 751-122KO cells.

### Interaction of miR-122 with G28A mutation changes the balance between translation and replication

**Fig 11:**
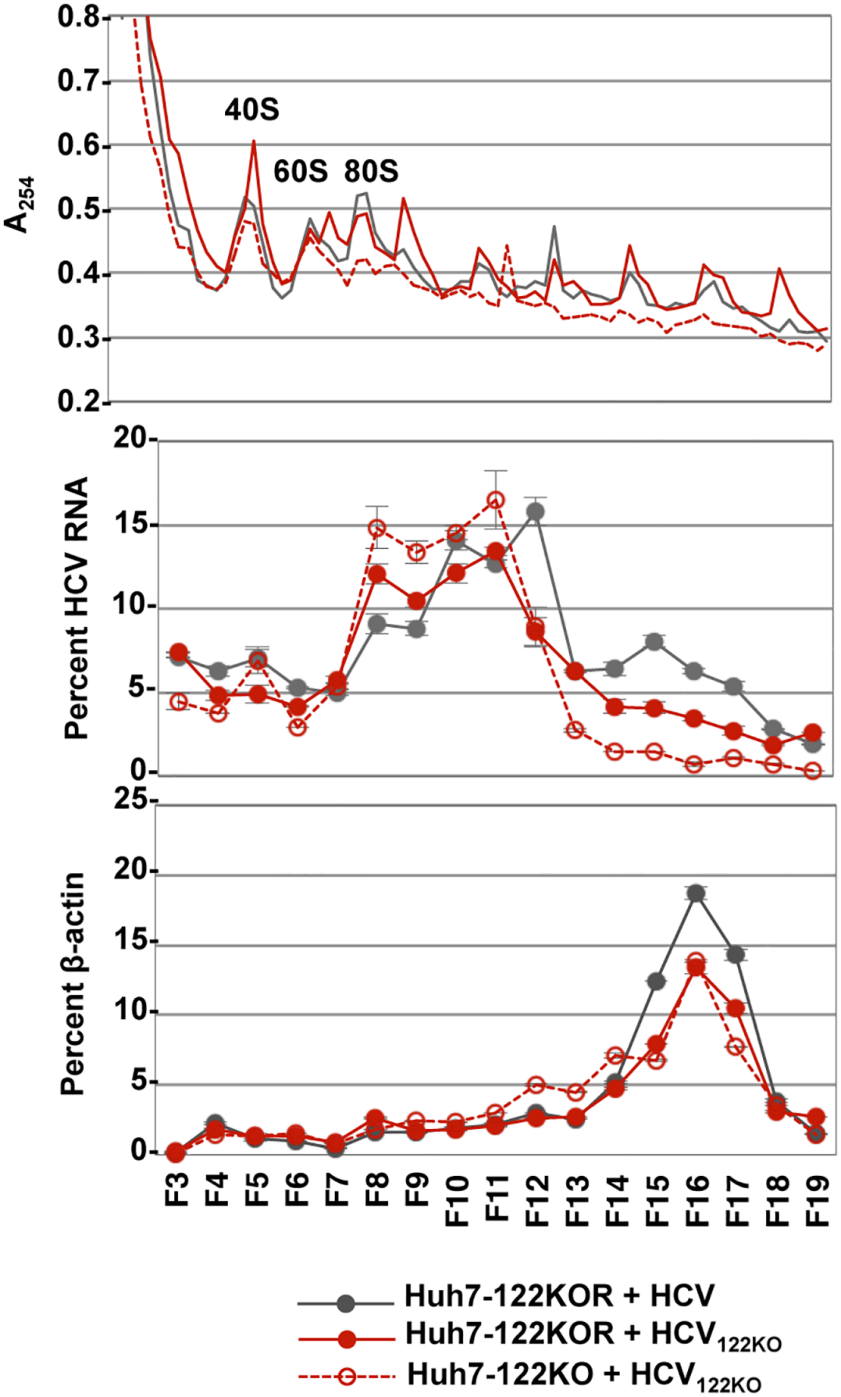
Polysome analysis of lysates from HCV- or HCV_122KO_-infected cells. Huh7 cells (5x10^5^ cells) were infected with HCV or HCV_122KO_ and harvested at 72 hpi for polysome analysis. A254 absorbance (top), distribution of HCV-RNA (middle) and β-actin mRNA levels (bottom) were determined.

We observed that replication complex formation was enhanced by G28A mutation, but propagation of HCV_122KO_ was comparable with that of wild type HCV in parental Huh7 cells (Fig 8D). Recently, it has been reported that miR-122 plays a role that rebalances RNA state from protein synthesis to replication by displacing PCBP2 from viral RNA [17]. To further examine the balance between translation and replication of HCV_122KO_, we analyzed the distribution of polysome in cells infected with either HCV or HCV_122KO_. Lysates of Huh7-122KO and Huh7-122KOR cells infected with either HCV or HCV_122KO_ were fractionated by a sucrose gradient. The proportion of polysome-free HCV-RNA was higher in Huh7-122KOR cells infected with HCV_122KO_ than in those infected with the wild type (Fig 11; fractions 7–9), suggesting that G28A mutation promotes replication rather than translation. Although miR-122 has been reported to enhance replication [17], the proportion of polysome-free HCV-RNA was higher in Huh7-122KO cells than Huh7-122KOR cells upon infection with HCV_122KO_. These results suggest that G28A mutation facilitates HCV propagation by enhancing formation of the replication complex under an miR-122-deficient condition and by fine-tuning the balance between replication and translation under an miR-122-abundant condition.

## Discussion

HCV exists as a quasispecies due to the low fidelity of its RNA-dependent RNA polymerase, and escape mutants often appear during treatment with anti-HCV drugs. On the other hand, miR-122 interaction sites in the 5’UTR of HCV-RNA are highly conserved. A previous report has shown that the administration of an miR-122 inhibitor to chimpanzees chronically infected with HCV achieved a long-term suppression of viral load without the emergence of a resistant virus or any adverse effects [43]. In addition, a recent phase II clinical trial of an miR-122 inhibitor revealed that it suppressed HCV propagation in chronic hepatitis C patients [26]. However, several studies have reported that adaptive mutations can be introduced around miR-122 interaction sites [44] and substitution of G28A in the 5’UTR of HCV has been selected by serial passages in miR-122-knockdown cells [27] and knockout cells [45]. In this study, we could also obtain an adaptive mutant with G28A mutation in the 5’UTR by adaptation to miR-122 knockout cells, HCV_122KO_, which was capable of propagating efficiently in miR-122-deficient cells, and analyzed its biological significance.

Most of the HCV genotypes, including genotype 1, originally have adenine at the position corresponding to G28A of genotype 2a. Although Israelow *et al*. showed that A28 genotypes have lower sensitivity to an miR-122 inhibitor than G28 genotypes [27], the emergence of adaptive mutation involved in the resistance to miR-122 inhibitor has been reported by the study about gt1b HCV. The C3U mutation adjacent to the miR-122 binding site I in the 5’UTR of the genotype 1b genome introduced during treatment with an miR-122 inhibitor in clinical practice has been shown to be resistant to the miR-122 inhibitor [38, 39]. Interestingly, in the current study, adaptive mutation C30U was introduced in the 5’UTR of Con1C3/JFH_122KO_, which has 5’UTR of genotype 1b. Although there is a difference of genome structure between the full-length viral genome and chimeric virus Con1C3/JFH used in our study, adaptive mutations were introduced in miR-122-binding sites. Together with the requirement of several substitutions throughout the genomic RNA of Con1C3/JFH_122KO_ to increase infectivity, these findings suggest that the adaptive mutations required not only for higher replication fitness but also for miR-122-independence such as G28A substitution of gt2a and C3U or C30U of gt1b should be introduced into the HCV genome to allow the virus to replicate efficiently under an miR-122-deficient condition. To examine the role of adaptive mutation C3U or C30U for miR-122-independence in gt1b HCV, further investigation is needed.

Based on our previous and current experiments, chronic HCV infection might induce viral replication not only in the liver but also in other non-hepatic tissues [28, 46] such as PBMCs and neuronal cells [3, 4]. The current study shows that during persistent infection in non-hepatic PBMCs of HCV gt2a patients, G28A mutation can be introduced into HCV-RNA, in some cases resulting in EHMs such as hypothyroidism and malignant lymphoma. G28A mutation can spontaneously emerge in non-hepatic cells such as PBMCs upon establishment of persistent infection. Once G28A mutation is introduced into viral genome, mutant viruses can replicate efficiently through an enhancement of replication complex formation without interaction of Ago2/miR-122 with HCV-RNA in non-hepatic cells. Most recently, several studies about HCV and lymphoma were reported. Lymphotropic HCV strain (HCV-SB) was isolated from HCV gt2b-positive B cell lymphoma with adaptive mutations in 5’UTR and E1/E2 region and B7.2 (CD86) was identified as a lymphotropic HCV receptor [47]. Moreover, after HCV treatment with DAA (sofosbuvir) and ribavirin to HCV gt3a patient, remission of lymphoma was induced [48]. These observations suggest that low levels of HCV-RNA replication in the non-hepatic tissues or liver with treatment of miR-122 inhibitors may persist in an miR-122-independent manner, resulting in the emergence of HCV mutants that might induce EHMs. Therefore, it is important to control the emergence of miR-122 inhibitors-resistant mutants, which confers a replicative advantage to HCV propagation in miR-122 deficient cells.

It has been shown that P-body components consisting of Xrn1, Lsm1 and PatL1, which are involved in mRNA decay, participate in translation of the HCV genome [49]. Although previous siRNA-mediated knockdown studies showed that miR-122 contributes to stabilization of the HCV genome by protecting it from exonuclease Xrn1 or Xrn2 [23, 24], HCV-RNA replication was not affected by Xrn1 knockdown in Huh7 cells [23], and the ability of miR-122 to promote HCV infectivity was shown to be independent of the protection of viral RNA from Xrn1 by a reconstituted *in vitro* system [50]. In this study, we observed no significant effect of Xrn1 and Xrn2 on the emergence of G28A mutation, suggesting that HCV-RNA decay by exonuclease was not involved in G28A-mediated miR-122-independence.

On the other hand, Lsm1 was suggested to be involved in the miR-122-dependent enhancement of translation of HCV-RNA in Huh7 cells [51]. Bromo mosaic virus, a single-stranded positive-sense RNA virus, requires the Lsm complex for switching from primary translation to viral RNA replication via viral protein-mediated trafficking of viral RNA to the ER [52]. Similarly, a recent study showed the importance to the HCV life cycle of a switching system in which miR-122 stimulates RNA synthesis by altering the balance of replication and translation [17]. PCBP2, which binds to the stem-loop I in the 5’UTR of HCV [53], is predicted to be required for the circularization of viral RNA through direct binding to the 5’ and 3’ ends of viral RNA, but this interaction was inhibited by miR-122 [54] followed by a switch from translation to viral RNA replication [17]. These reports suggest that the inefficient replication of HCV in miR-122-deficient cells may be attributable to degradation of viral RNA and a delay in the switching from translation to replication. We have shown that HCV replicon RNA possessing G28A mutation exhibits efficient colony formation in the absence of miR-122. In addition, a competition assay revealed that HCV_122KO_ carrying a mutation of G28A in the 5’UTR exhibits a growth advantage compared to wild type HCV in the absence but not in the presence of miR-122, suggesting that G28A mutation in the 5’UTR of HCV confers at least limited ability to propagate under an miR-122-deficient condition. Polysome analysis clearly showed the excessive replication status in the G28A mutant in miR-122-deficient cells, resulting in efficient propagation. Although a system for equilibrating PCBP2 and the miR-122-Ago2 complex exists, the role of miR-122 on the G28A mutant is to moderate excessive replication but enhance translation, which is the opposite of its role on the wild type virus. These observations indicate that miR-122 may fine-tune the balance between translation and replication not only in the wild type but also the G28A mutant and thereby lead to efficient propagation.

In summary, we have shown that HCV can replicate in an miR-122-independent manner. This finding indicates that treatment of hepatitis C patients with an inhibitor for miR-122 would be effective to eliminate HCV in the liver but not in non-hepatic tissues, and may induce the emergence of adaptive mutants capable of propagating in miR-122-deficient cells, leading to the induction of EHMs.

## Materials and methods

### Plasmids

cDNA clones of wild type miR-122 (WT-miR-122) and AcGFP were inserted between the XhoI and XbaI sites of a lentiviral vector, pCSII-EF-RfA, which was provided by M. Hijikata, and the resulting plasmids were designated pCSII-EF-WT-miR-122 and pCSII-EF-AcGFP, respectively. pHH-JFH1 and pJFH1 encoding full-length cDNA and pSGR-JFH1 encoding subgenomic cDNA of the JFH1 strain [55, 56] were kindly provided by T. Wakita. pHH-JFH1-E2p7NS2mt and pJFH1-E2p7NS2mt contain three adaptive mutations in pHH-JFH1 and pJFH1 [57], respectively. pCon1/C3/JFH1, which encodes Con1/JFH1 (genotype 1b/2a) chimeric virus genome, was fused at the first transmembrane domain of NS2 (C3; described by Pietschmann et al. [58]). pmirGLO-1 (pmirGLO-compl-miR-122) was described previously [28]. The complementary sequences of miR-122-5p were introduced into the multicloning site of the pmirGLO vector (Promega), and the resulting plasmid was designated pmirGLO-2. The plasmid pX330 (Addgene plasmid 42230) designed for the CRISPR-Cas9 system [59, 60] was provided by Addgene. The plasmids used in this study were confirmed by sequencing with an ABI PRSM 3130 genetic analyzer (Life Technologies, Tokyo, Japan).

### Cells

All cell lines were cultured at 37°C under the conditions of a humidified atmosphere and 5% CO_2_. Human hepatocellular carcinoma cell line Huh7, human embryonic kidney cell line 293T and human endometrial adenocarcinoma cell line Hec1B were obtained from Japanese Collection of Research Bioresources (JCRB) Cell Bank (JCRB0403, JCRB9068 and JCRB1193). Human lung squamous-cell carcinoma cell line RERF-LC-AI cells was provided by the RIKEN BRC through the National Bio-Resource Project of MEXT, Japan (RCB0444). The human lung adenocarcinoma epithelial cell line A549, human renal cell carcinoma cell line Caki-2 and human neuroepithelioma cell line MC-IXC were obtained from the American Type Culture Collection (ATCC CCL-185, ATCC HTB-47, and CRL-2270). The Huh7-derived hepatocellular carcinoma cell line Huh7.5.1 was provided by F. Chisari. Human marrow stromal cells (HMSCs) were obtained from Cell Applications Inc. (San Diego, CA). Except for HMSC, all cell lines were maintained in Dulbecco’s modified Eagle’s medium (DMEM) (Sigma, St. Louis, MO) supplemented with 100 U/ml penicillin, 100 μg/ml streptomycin, and 10% fetal bovine serum (FBS). HMSCs were maintained in MF-medium with 1% FBS (Toyobo, Japan). Replicon cell lines harboring HCV-RNA were maintained in DMEM containing 10% FBS and 1 mg/ml G418 (Nakalai Tesque, Kyoto, Japan).

### Viruses

pHH-JFH1-E2p7NS2mt was transfected into Huh7.5.1 cells, and the culture supernatants were collected after serial passages. Infectivity of HCV was determined by focus-forming assay and expressed in focus-forming units (FFU) [56]. Unless otherwise noted, cells were infected with HCV at an MOI of 1. The lentiviral vectors and ViraPower Lentiviral Packaging Mix (Life Technologies, San Diego, CA) were co-transfected into 293T cells and the supernatants recovered at 48 h post-transfection were centrifuged at 1000 x g for 5 min and cleared through a 0.45 μm filter. The infectious titer of lentivirus was determined by a Lenti-X qRT-PCR Titration Kit (Clontech, Mountain View, CA). The vesicular stomatitis virus (VSV) variant NCP12.1, derived from the Indiana strain, was provided by M. Whitt. The pseudotype VSVs bearing the HCV E1 and E2 glycoproteins (HCVpv) and VSV G protein (VSVpv) were prepared as described previously [61]. Infectivity of the pseudotype viruses was assessed by the expression of luciferase as determined by a Bright-Glo Luciferase assay system (Promega), following a protocol provided by the manufacturer and expressed in relative light units (RLU).

### Clinical samples

Thirty-six HCV gt2a patients being treated at Hiroshima University Hospital were enrolled in this study. PBMCs were isolated using Ficoll-Hypaque density gradient centrifugation from a patient.

### Ethics statement

The study was approved by the Ethical Committee of the Research Institute for Microbial Diseases, Osaka University and Hiroshima University of Medicine. Written informed consent was obtained from all enrolled patients.

### Antibodies and drugs

Mouse monoclonal antibodies to HCV NS5A and β-actin were purchased from Austral Biologicals (San Ramon, CA) and Sigma-Aldrich, respectively. Rabbit anti-HCV core antibody was prepared as described previously [62]. Mouse monoclonal antibodies to HCV core (C7-50) were purchased from Thermo Fisher Scientific (Waltham, MA). Anti-human CD81 (hCD81) monoclonal antibody (JS-81) was purchased from BD Biosciences (Franklin Lakes, NJ). Mouse anti-double-stranded RNA (dsRNA) IgG_2a_ (J1 and K2) antibodies were from English and Scientific Consulting Kft. (Szirak, Hungary). Mouse anti-IgG was purchased from Jackson ImmunoResearch (West Grove, PA). Mouse monoclonal anti-Ago2 was purchased from Abcam (Cambridge, UK). Rabbit polyclonal anti-Xrn1 and anti-scavenger receptor class B type1 (SR-BI) antibodies were purchased from Novus Biologicals (Littleton, CO). Rabbit polyclonal anti-Xrn2 was purchased from Proteintech (Rosemont, IL). Rabbit anti-CLDN1 and anti-occludin (OCLN) antibodies, Alexa Fluor (AF) 488-conjugated anti-rabbit IgG, AF594-conjugated anti-mouse IgG and rabbit anti-CLDN1 antibodies were from Life Technologies. Mouse anti-apolipoprotein E (ApoE) antibody was purchased from Santa Cruz (Santa Cruz, CA). Human recombinant IFNα was purchased from PBL Biomedical Laboratories (Piscataway, NJ). The HCV NS3-4A protease inhibitor BILN was purchased from Acme Bioscience (Salt Lake City, UT). The NS5A inhibitor BMS-790052 and NS5B inhibitor PSI-7977 were purchased from Shanghai Haoyuan Chemexpress (Shanghai, China). Anti-CD81 antibody (5 ng/ml) was pre-treated before HCV infection and IFNα (100U/ml), BILN (0.5 μM), BMS-790052 (0.5 nM) and PSI-7977 (100 nM) were treated at 2 h post-transfection. BODIPY 558/568 lipid probe and DAPI (4’, 6-diamidino-2-phenylindole) were purchased from Life Technologies and Vector Laboratories Inc. (Burlingame, CA), respectively. Locked nucleic acids (LNA) complementary to miR-122 (5′-CcAttGTcaCaCtCC-3′; LNA-miR-122) and its negative control (5’-CcAttCTgaCcCtAC-3’; LNA-Cont) (LNA in capitals, DNA in lowercase; sulfur atoms in oligonucleotide phosphorothioates are substituted for non-bridging oxygen atoms; capital C indicates LNA methylcytosine) [63] were purchased from Gene Design (Osaka, Japan) and transfected into cells using Lipofectamine RNAi MAX (Life Technologies) according to the manufacturer’s protocol (reverse transfection protocol). The miScript miRNA mimic hsa-miR-122 and its negative control were purchased from Qiagen (Valencia, CA).

### Quantitative RT-PCR

Total RNA was prepared from cells by using an RNeasy mini kit (Qiagen). For quantitation of HCV-RNA, quantitative RT-PCR was performed by using TaqMan EZ RT-PCR Core Reagents and a ViiA7 system (Life Technologies) according to the manufacturer’s protocol. For quantitation of gene expression, the synthesis of the first-stranded cDNA was performed by using a PrimeScript RT Reagent Kit (Perfect Real Time) (Takara Bio) and quantitative RT-PCR was performed by using Platinum SYBR Green qRT-PCR SuperMix UDG (Life Technologies) according to the manufacturer’s protocol. ApoB, ApoE and MTTP were amplified using the primer pairs described previously [28]. For quantitation of miRNA, total RNA was prepared from cells by using an miRNeasy mini kit (Qiagen) and miR-122 was determined by using miR-122-specific RT primers and amplified by using specific primers provided in the Taqman MicroRNA Assays (Life Technologies) according to the manufacturer’s protocol. U6 small nuclear RNA (snRNA) was used as an internal control. Fluorescent signals were analyzed by using a ViiA7 system (Life Technologies).

### Transfection and immunoblotting

Cells were transfected with the plasmids by using *Trans* IT LT-1 transfection reagent (Mirus, Madison, WI) according to the manufacturer’s protocols. The cells were lysed on ice in Triton lysis buffer (20 mM Tris-HCl (pH 7.4), 135 mM NaCl, 1% Triton-X 100, 10% glycerol) supplemented with a protease inhibitor mix (Roche). The samples were boiled in loading buffer and subjected to 5–20% gradient sodium dodecyl sulfate-polyacrylamide gel electrophoresis (SDS-PAGE). The proteins were transferred to polyvinylidene difluoride membranes (Millipore, Bedford, MA), and reacted with primary antibody and then secondary horseradish peroxidase-conjugated antibody. The immunocomplexes were visualized with Super Signal West Femto substrate (Pierce, Rockford, IL) and detected by using an LAS-3000 image analyzer (Fujifilm, Tokyo, Japan).

### Knockout of miR-122 by TALEN in Huh7 or Huh7.5.1 cells

Left and Right Custom TALEN (Cellectis, Paris, France) targeting miR-122 seed sequences were designed by TALEN Hit Search (TTCCTTAGCAGAGCTGTGGAGTGTGACAATGGTGTTTGTGTCTAAACTA; the TALEN targeting sequence is underlined and the left or right TAL effector DNA-binding domains are on either side of the target), and the corresponding expression plasmids were purchased from Takara Bio (Shiga, Japan). Two TALEN targeting miR-122 RNAs were synthesized by using an mMESSAGE mMACHINE T7 Ultra kit (Life Technologies) according to the manufacturer’s protocol. Huh7 cells and Huh7.5.1 cells were seeded onto 6-well plates at the concentration of 2x10^5^ cells/well and transfected with 2 μg of each of the Custom TALEN RNAs using Lipofectamine 2000 (Life Technologies). The culture medium was replaced with fresh DMEM containing 10% FBS at 4 h post-transfection, incubated at 30°C for 48 h and then shifted to 37°C. When the cells became confluent, single cells were seeded onto 96-well plates and total DNA was isolated from each clone after it had grown to maturity. PCR products amplified by using a primer set (miR-122-F: 5’-CAAGATGCTTGTACCCGTGA-3’; miR-122-R: 5’-GTGCCTGGTCTGCAATCTTT-3’) were denatured, annealed, and then treated with SURVEYOR (Transgenomic) nuclease or sequenced after cloning into a pGEM-Teasy vector (Promega).

### Knockdown of Xrn1 and Xrn2 by shRNA expression system by lentiviral vector in Huh7.5.1 cells

A lentiviral vector that expresses a short hairpin RNA (shRNA) was generated as previously reported [64] by using pFTRE3G_pGK_GFP [65]. Briefly, the U6 promoter and pGK_Hygro cassette were inserted between PacI and AscI sites of pFTRE3G_pGK_GFP and the resulting plasmid was designated FU6_pGKhygro. To knockdown the Xrn1 and Xrn2 genes, oligonucleotides (shXrn1-s: 5’-GATCCGAGGTGTTGTTTCGCATTATTCAAGAGATAATGCGAAACAACACCTCTTTTTTG-3’; shXrn1-as: 5’-AATTCAAAAAAGAGGTGTTGTTTCGCATTATCTCTTGAATAATGCGAAACAACACCTCG-3’, shXrn2-s: 5’-GATCCGAACCGAACTTTACCATTATTCAAGAGATAATGGTAAAGTTCGGTTCTTTTTTG-3’; shXrn2-as: 5’-AATTCAAAAAAGAACCGAACTTTACCATTATCTCTTGAATAATGGTAAAGTTCGGTTCG-3’) were annealed and inserted into the EcoRI and BamHI sites of the FU6_pGKHygro vector, respectively, and used for generating lentivirus. 751-122KO#1 cells were infected with lentivirus and then cultured in DMEM with 200 μg/ml Hygromycin. Each cell clone was used for Western blotting.

### Indirect immunofluorescence assay

Cells cultured on glass slides were fixed with 4% paraformaldehyde in phosphate buffered saline (PBS) at room temperature for 30 min. After washing three times with PBS, the cells were permeabilized for 20 min at room temperature with PBS containing 0.2% Triton-X and blocked with phosphate buffer containing 2% bovine serum albumin (BSA) for 1 h at room temperature. The cells were incubated with blocking buffer containing rabbit anti-NS5A or rabbit anti-core at room temperature for 1 h, washed three times with PBS and incubated with blocking buffer containing appropriate AF488-conjugated and AF594-conjugated secondary antibodies at room temperature for 1 h. Finally, the cells were washed three times with PBS and observed with a FluoView FV1000 laser scanning confocal microscope (Olympus, Tokyo, Japan).

### Cell growth

Cell growth was determined by the Cell Titer-Glo Luminescent Cell Viability Assay (Promega) according to the manufacturer’s protocol and expressed in RLU at 24, 48 and 72 h post-seeding.

### Sample collection and RNA extraction for microarray analysis

Total RNAs extracted from cells were purified by using an miRNeasy Kit (Qiagen, Valencia, CA) according to the manufacturer’s protocol. Eluted RNAs were quantified using a Nanodrop ND-1000 v3.5.2 spectrophotometer (Thermo Scientific, Wartham, MA). RNA integrity was evaluated using the RNA 6000 LabChip and Bioanalyzer (Agilent Technologies, Santa Clara, CA). Each RNA that had an RNA integrity number (RIN) greater than 9.0 was used for the microarray experiments.

### Microarray experiment

Expression profiling was generated using the 4 x 44 K whole human genome oligo-microarray ver. 2.0 G4845A (Agilent Technologies). Each microarray uses 44,495 probes to interrogate 27,958 Entrez gene RNAs. One-hundred nanograms of total RNA were reverse-transcribed into double-strand cDNAs by AffinityScript multiple-temperature reverse-transcriptase and amplified for 2 h at 40°C. The resulting cDNAs were subsequently used for *in vitro* transcription by the T7 RNA polymerase and labeled with cyanine-3-labeled cytosine triphosphate (Perkin Elmer, Waltham, MA) for 2 h at 40°C using a Low Input Quick-Amp Labeling Kit (Agilent Technologies) according to the manufacturer’s protocol. After labeling, the rates of dye incorporation and quantification were measured with a Nanodrop ND-1000 v3.5.2 spectrophotometer (Thermo Scientific) and then were fragmented for 30 min at 60°C in the dark. Differentially labeled samples of 1650 ng of cRNA were hybridized on Agilent 4 x 44K whole genome arrays ver. 2.0 (Agilent Design #026652) at 65°C for 17 h with rotation in the dark. Hybridization was performed using a Gene Expression Hybridization Kit (Agilent Technologies) following the manufacturer’s instructions. After washing in GE washing buffer, each slide was scanned with an Agilent Microarray Scanner G2505C. Feature extraction software (Version 10.5.1.1) employing defaults for all parameters was used to convert the images into gene expression data. Raw data were imported into Subio platform ver.1.12 (Subio) for database management and quality control. Raw intensity data were normalized against GAPDH expression levels for further analysis. These raw data have been accepted by GEO (a public repository for microarray data, aimed at storing MIAME [Minimum Information About Microarray Experiment]). Access to data for this study may be found under GEO experiment accession number GSE32886.

### Electron microscopy and correlative fluorescence microscopy-electron microscopy (FM-EM) analysis

Cells were cultured on a Cell Desk polystyrene coverslip (Sumitomo Bakelite, Osaka, Japan), and were fixed with 2% formaldehyde and 2.5% glutaraldehyde in 0.1 M cacodylate buffer (pH 7.4) containing 7% sucrose. Cells were post-fixed for 1 h with 1% osmium tetroxide and 0.5% potassium ferrocyanide in 0.1 M cacodylate buffer (pH 7.4), dehydrated in a graded series of ethanol and embedded in Epon812 (TAAB). Ultrathin (80 nm) sections were stained with saturated uranyl acetate and lead citrate solution. Electron micrographs were obtained with a JEM-1011 transmission electron microscope (JEOL, Tokyo, Japan). Correlative FM-EM allows individual cells to be examined both in an overview with FM and in a detailed subcellular-structure view with EM. The NS5A was stained and observed in the HCV-infected cells by the correlative FM-EM method as described previously [66].

### *In vitro* transcription, RNA electroporation and colony formation

The plasmids pSGR-JFH1, pJFH1-E2p7NS2mt and pCon1/C3/JFH1 were linearized with XbaI and then treated with Mung bean exonuclease. The linearized DNAs were transcribed *in vitro* by using the MEGAscript T7 kit (Life Technologies) according to the manufacturer’s protocol. The *in vitro* transcribed RNA (5 μg) was electroporated into each cell at 5x10^6^ cells/0.4 ml under conditions of 190V and 950 μF using a Gene Pulser (Bio-Rad, Hercules, CA) and plated on DMEM containing 10% FBS. For colony formation, the medium was replaced with fresh DMEM containing 10% FBS and 1 mg/ml G418 at 24 h post-electroporation. The remaining colonies were cloned by using a cloning ring (Asahi Glass, Tokyo, Japan) or fixed with 4% paraformaldehyde and stained with crystal violet at 21 days post-electroporation.

### Immunoprecipitation

Cells were suspended with IP buffer (25 mM Tris-HCl (pH 7.4), 150 mM KCl, 5 mM EDTA, 5 mM DDT, RNase inhibitor (100 U/ml), protease inhibitor (Roche)) and placed on ice for 15 min, followed by sonication for 2 min. Cell lysates were collected and centrifuged for 10 min at 2,000 rpm at 4°*C*. Supernatants were preincubated with Protein G Sepharose (GE Healthcare) for 1 h and anti-Ago2 or control IgG at 4°C for 2 h followed by addition of 30 μl of Protein G Sepharose (GE Healthcare) for 1 h. The Sepharose beads were washed three times in PBS and RNAs were extracted using the Qiazol reagent (Qiagen). HCV-RNA associated with Ago2 protein was detected by qRT-PCR as described as above.

### Northern blotting

For detection of miRNA, total RNA was prepared from cells by using an miRNeasy mini kit (Qiagen) and samples were subjected to 15% TBE-Urea polyacrylamide gel electrophoresis after boiling in loading buffer. The total RNAs were transferred to nylon membranes (Roche, Mannheim, Germany), and miR-122 was detected by using an miR-122-specific 5’- digoxigenin (DIG)-labeled miRCURY LNA detection probe (Exiqon, Vedbaek, Denmark) and visualized with a DIG luminescence detection kit (Roche) according to the manufacturer’s protocol.

### Deep sequencing analysis

RNA was extracted from 100 μl of virus-containing culture supernatants after 5 passages in Huh7.5.1 cells (JFH-P5) or a mixture of those after 5 and 4 passages in 751-122KO#1 and 751-122KO#2 cells, respectively (6 x 122KO). The first-stranded cDNA was synthesized by using a PrimeScript 1st strand cDNA Synthesis Kit (Takara Bio) and three fragments of the HCV genome region were amplified. PacBio DNA libraries were prepared from three pooled fragments (each 100 ng) of the respective HCV genomes using a DNA Template Prep Kit 2.0 (3–10 kbp) (Pacific Biosciences) according to the manufacturer’s instructions. Sequencing was performed by the PacBio RS II system with a 240 min movie using the DNA Sequencing Kit 4.0 (Pacific Biosciences) with P6 polymerase. Circular consensus sequences (CCS) constructed from more than four full-pass subreads were produced through PacBio SMRT analysis.

### Analysis of the 5’UTR sequence of HCV

For a rapid identification of 5’UTR sequence of HCV, RNA was extracted from 100 μl of virus-containing supernatants or PBMCs. The first-stranded cDNA was synthesized by using a PrimeScript RT reagent Kit (Perfect Real Time) (Takara Bio) and the 5’UTR of HCV was amplified. 5’RACE was performed by using a 5'RACE System for Rapid Amplification of cDNA Ends, Version 2.0 (Life Technologies) as described by Li et al. [44] with modification (see S2 Table).

### Polysome analysis

Polysome analysis was performed as described by Masaki et al. [17] with some modifications. In brief, three 10-cm dishes containing 5x10^5^ of Huh7-122KO or Huh7-122KOR cells were infected with HCV or HCV_122KO_. At 3 dpi, the cells were incubated with CHX (100 μg/ml) for 10 min. After washing with PBS with CHX (100 μg/ml), the cells were harvested from three dishes (total number of cells: ~2x10^7^ cells/dish) by using a cell scraper and centrifuged for 5 min at 1,400 rpm at 4°C. Cell pellets were suspended in 500 μl of polysome lysis buffer (PLB; 140 mM KCl, 5 mM MgCl_2_, 20 mM Tris-HCl (pH 7.4), 0.01% Triton X-100, 10 mM DTT, 100 μg/ml CHX) with RNase inhibitor (100 U/ml) and passaged 5 times with a 27-gauge needle on ice. Cell pellets were removed by two 5-min rounds of centrifugation at 13,000 rpm and 4°C. The supernatant was layered on the top of a linear 10%-50% sucrose gradient in PLB and centrifuged in an SW41Ti rotor (Beckman Coulter, CA, USA) for 2 h at 32,000 rpm at 4°C (no brake). The absorbances at OD254 of the 76 fractions collected from the top by using a Piston Gradient Fractionator (BioComp, NB, Canada) were determined and divided into 19 fractions for quantification of HCV-RNA and β-actin mRNA by qRT-PCR as described above.

### Statistical analysis

The data for statistical analyses are the average of three independent experiments. Results were expressed as the means ± standard deviation. The significance of differences in the means was determined by Student’s *t*-test.

## Supporting information

S1 Fig Establishment of miR-122-knockout Huh7 cells. (A) A PCR product (450 bp) including a TALEN-targeted miR-122 seed region was digested with Cel-I, resulting in 300 and 150 bp fragments (red arrowheads). We obtained 2 clones of miR-122-knockout Huh7 cell clones #1 and #2. (B) The target sequence of TALEN for knockout of miR-122 and the genome sequence of the miR-122 allele in Huh7-122KO cells. Mutations in the miR-122 allele with an 11 nt or a 21 nt deletion in the Huh7-122KO#1 clones or a 2 nt or an 8 nt deletion in the Huh7-122KO#2 clones were identified. (C) Detection of miR-122 expression by Northern blot (top panel) and qRT-PCR (bottom). Total RNA was extracted from each cell and the relative expression of miR-122 was determined by qRT-PCR by using U6 snRNA as an internal control. (D) miR-122 activity in miR-122-knockout Huh7 cells. pmirGLO vectors carrying the complementary sequence of miR-122 under the luciferase gene were transfected into Huh7-122KO and Huh7-122KOR cells. At 48 h post-transfection, the luciferase activity was determined. The data are representative of three independent experiments. Error bars indicate the standard deviation of the mean and asterisks indicate significant differences (**P < 0.01) versus the results for the control.
(TIF)

S2 Fig Knockout of the miR-122 gene from Huh7 cells exhibits no significant effect on cell growth. The effect of miR-122 knockout on cell growth was determined by using a Cell Titer-Glo Luminescent Cell Viability Assay. Equal amounts of Huh7-122KO#1 and Huh7-122KOR#1 cells were seeded and RLU were determined at 24, 48, and 72 h post-seeding.
(TIF)

S3 Fig Knockout of the miR-122 gene from Huh7 cells exhibits no significant effect on the entry of pseudotyped VSV bearing HCV envelope proteins. Entry of pseudotyped VSVs bearing no envelope proteins or the HCV and VSV envelope proteins, GFPpv, HCVpv, and VSVpv, respectively, into Huh7, Huh7-122KO, and Huh7-122KOR cells. Luciferase activity was determined at 24 h post-infection.
(TIF)

S4 Fig Knockout of the miR-122 gene from Huh7 cells exhibits no significant effect on the replication of HCV SGR RNA. (A) Huh7-122KO-SGR and Huh7-122KOR-SGR cells were fixed with 4% PFA and stained with anti-NS5A antibody (green) and BODIPY for lipid droplets (red). Cell nuclei were stained with DAPI (blue). (B) Electron microscopy of Huh7-122KO-SGR and Huh7-122KOR-SGR cells. The boxes in the lower panels were magnified and the red arrows indicate membranous web-like structures.
(TIF)

S5 Fig Treatment of Huh7-122KO-SGR cells with IFNα and HCV NS3-4A inhibitor. Intracellular HCV-RNA in Huh7-122KO-SGR #1, #3 or #5 cells treated with a combination of 100 IU/ml of IFN-α and 200 nM of the NS3-4A protease inhibitor BILN was quantified by qRT-PCR at 36 hpi. Error bars indicate the standard deviation of the mean and asterisks indicate significant differences (**P < 0.01) versus the results for the control.
(TIF)

S6 Fig miR-122-independent propagation of HCV in miR-122 KO cells. Full-genomic HCV-RNA of the JFH1 strain was electroporated into Huh7-122KO cells together with either the control- or miR-122-mimic, and then the infectious titers in the culture supernatants were determined at 3, 6, 9, 12, 24, 36, 48, and 60 h post-electroporation (hpe).
(TIF)

S7 Fig Co-localization of NS5A and membrane structures in Huh7-122KO cells. HCV NS5A in Huh7-122KO cells was observed by the FM-EM method. The boxes (1 and 2) in the right top panel were magnified (bottom), respectively.
(TIF)

S8 Fig Co-localization of HCV core proteins and lipid droplets in Huh7-122KO cells. Huh7-122KO and Huh7-122KOR cells infected with HCV and those mock-infected were fixed at 72 hpi and stained with antibodies to core protein (green) and BODIPY for lipid droplets (red). Cell nuclei were stained with DAPI (blue).
(TIF)

S9 Fig Expression levels of apoE, apoB and MTTP were decreased in Huh7-122KO cells. The expression levels of apoE, apoB and MTTP in Huh7-122KO and Huh7-122KOR cells were analyzed by qRT-PCR. Error bars indicate the standard deviation of the mean and asterisks indicate significant differences (*P < 0.05; **P < 0.01) versus the results for the control.
(TIF)

S10 Fig miR-122 exhibits no significant effect on particle formation of HCV. Specific infectivity (infectious titers/intracellular RNA copies) was calculated at 72 h post-infection.
(TIF)

S11 Fig Establishment of miR-122KO Huh7.5.1 (751-122KO) cells and efficient propagation of HCV. (A) Target sequence of TALEN for knockout of miR-122 and genome sequence of the miR-122 allele in 751-122KO cells. A 10 nt insertion into a 7 nt deletion and an 11 nt deletion, 50 nt and 11 nt deletions, and 7 nt and 22 nt deletions in the miR-122 allele were observed in 751-122KO#1, 751-122KO#2, and 751-122KO#3 cells, respectively. (B) The relative expression of miR-122 was determined by qRT-PCR.
(TIF)

S12 Fig Establishment of cured Huh7-122KO cells. Elimination of HCV-RNA from Huh7-122KO#2-derived JFH-SGR cells. Two clones derived from Huh7-122KO-SGR cells (#3 and #5) were treated with a combination of 100 IU/ml of IFN-α and 200 nM of BILN to eliminate the HCV genome. The intracellular HCV-RNA level at each treatment (every 3 or 4 days) was determined by qRT-PCR.
(TIF)

S13 Fig Propagation of HCV_122KO_ in non-hepatic cells. HCV (black circles) and HCV_122KO_ (red circles) were inoculated into non-hepatic A549, Caki-2, MC-IXC and RERF-LC-AI cells at an MOI of 1 and intracellular HCV-RNA was determined by qRT-PCR at the indicated time points. Error bars indicate the standard deviation of the mean and asterisks indicate significant differences (*P < 0.05; **P < 0.01) versus the results for the control.
(TIF)

S14 Fig Propagation of HCV_122KO_ in HMSC cells. (A) Immunoblotting of SRBI, OCLN, CLDN1 and β-actin in primary HMSC cells exogenously expressing CLDN1 and OCLN. (B) HMSC cells and those expressing CLDN1 and OCLN were infected with either HCV or HCV_122KO_ and intracellular HCV-RNA was determined at 12, 24 and 48 hpi. Error bars indicate the standard deviation of the mean and asterisks indicate significant differences (**P < 0.01) versus the results for the control.
(TIF)

S15 Fig Inhibition of HCV and HCV_122KO_ replication in non-hepatic cells by the treatment with HCV NS3-4A inhibitor. Intracellular HCV-RNA in Huh7-122KO (top), Hec1B (middle) and 293T-CLDN1 cells (bottom) infected with either HCV or HCV_122KO_ were treated with NS3-4A protease inhibitor BILN, and then the intracellular HCV-RNA level was determined by qRT-PCR at 36 hpi. Error bars indicate the standard deviation of the mean and asterisks indicate significant differences (**P < 0.01) versus the results for the control.
(TIF)

S16 Fig Deep sequencing analysis. (A) 751-122KO#1 and #2 cells (each from three independent cultures) or Huh7.5.1 cells were infected with HCV at an moi of 10, the culture supernatants were collected after serial passages, and the infectious titers in the supernatants were determined by plaque assay. (B, C) Three fragments amplified from RNA purified from the culture supernatants after 5 passages in Huh7.5.1 cells (JFH-P5) or a mixture of those after 5 and 4 passages in 751-122KO#1 and 751-122KO#2 cells, respectively (6 x 122KO), were sequenced by deep sequencing analysis. The read coverage frequency (B) and the substitutions detected in 6 x 122KO (5% cut-off) (C) are shown.
(TIF)

S17 Fig Identification of mutations in the 5’UTR of HCV_122KO_. (A) pHH-JFH1-E2p7NS2mt was transfected into 751-122KO (#1, #2 and #3) cells, the culture supernatants were collected after serial passages, and infectious titers in the supernatants were determined by plaque assay. RNA purified from the culture supernatants after 5 and 4 passages in 751-122KO#1 and 751-122KO#2 cells, respectively (B), or Huh7-122KO-SGR#1 and Huh7-122KOR-SGR#1 cells (C) were sequenced. Arrows indicate the position of nt28 in the 5’UTR of JFH1-E2p7NS2mt and isolated viral RNA. Each RNA base is shown as a colored peak: A, green; U, red; G, black; and C, blue.
(TIF)

S18 Fig Replication kinetics of G28A virus and HCV_122KO_ in miR-122 KO cells. pJFH1-E2p7NS2mt or pJFH1-E2p7NS2mt-G28A RNA was electroporated into Huh7.5.1 cells, and the culture supernatants were collected at 4 dpi as G28 virus or G28A virus. Huh7.5.1 and 751-122KO#1 cells were infected with either G28 virus, G28A virus, HCV or HCV_122KO_ at an MOI of 1 and intracellular HCV-RNA and infectious titers in the supernatants were determined by qRT-PCR and focus formation assay, respectively, at the indicated time points. Error bars indicate the standard deviation of the mean and asterisks indicate significant differences (**P < 0.01) versus the results for the control.
(TIF)

S19 Fig Direct sequencing analysis of Con1C3/JFH and Con1C3/JFH_122KO_. Three amplified fragments of cDNA derived from Con1C3/JFH (A) or Con1C3/JFH_122KO_ (B) were sequenced by direct sequencing analysis. Black circles (non-synonymous substitution), white circles (synonymous substitution) and asterisks (single nucleotide mutation in 5’UTR) are shown at the position of each mutation on the diagram of the HCV-RNA structure. Only additional mutations were shown in Con1C3/JFH_122KO_ (B) compared to Con1C3/JFH. (C) RNA purified from Con1C3/JFH or Con1C3/JFH_122KO_ were sequenced, respectively. Arrows indicate the position of nt30 in the 5’UTR of isolated viral RNA. Each RNA base is shown as a colored peak: A, green; U, red; G, black; and C, blue.
(TIF)

S20 Fig Exonucleases are not invoved in the emergence of G28A mutation. (A) Establishment of Xrn1 or Xrn1/Xrn2 stable-knockdown miR-122 KO Huh7.5.1 cells. The results of immunoblotting of Xrn1, Xrn2 or β-actin from three clones each of 751-122KO-shlacZ, 751-122KO-shXrn1 or 751-122KO-shXrn1/Xrn2 cells (#1~#3) were shown. (B) Direct sequencing analysis of adapted viruses independently isolated in each of 751-122KO-shlacZ, 751-122KO-shXrn1 or 751-122KO-shXrn1/Xrn2 cells at passages 0 (virus stock) and 4 are shown. Arrows indicate the position of nt28 in the 5’UTR of HCV. Each RNA base is shown as a colored peak: A, green; U, red; G, black; and C, blue.
(TIF)

S21 Fig No revertant virus emerged during the passages of HCV_122KO_ in Huh7.5.1 cells. Huh7.5.1 cells were infected with HCV_122KO_ at a high or low titer and collected after eight serial passages. RNA purified from the culture supernatants was sequenced. Arrows indicate the position of nt28 in the 5’UTR of each isolated viral RNA. Each RNA base is shown as a colored peak: A, green; U, red; G, black; and C, blue.
(TIF)

S1 Table Pathway prediction in miR-122 KO cells by IPA. The top 10 predicted pathways generated by IPA from the change of gene expression by miR-122 knockout.
(TIF)

S2 Table Primers used for 5’RACE and rapid identification of G28A in JFH1 5’UTR or T302 in Con1C3/JFH 5’UTR. (TIF)
