# Supplementary figures and images for: Characterization of miR-122-independent propagation of HCV
Source: PLoS Pathog. 2017 May 11;13(5):e1006374. doi: 10.1371/journal.ppat.1006374 (PMC5441651; doi:10.1371/journal.ppat.1006374)

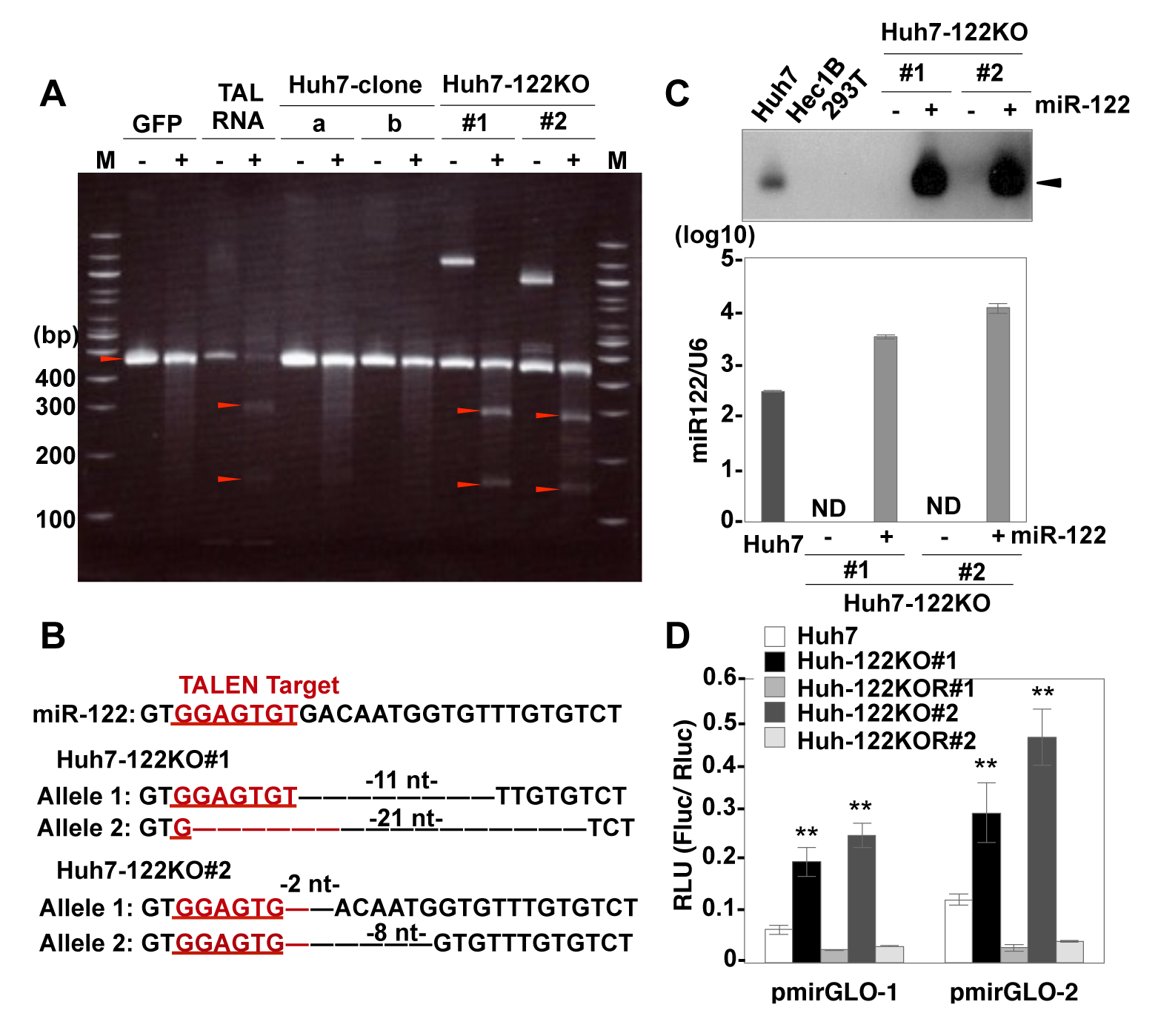

Supplement: S1 Fig — (A) A PCR product (450 bp) including a TALEN-targeted miR-122 seed region was digested with Cel-I, resulting in 300 and 150 bp fragments (red arrowheads). We obtained 2 clones of miR-122-knockout Huh7 cell clones #1 and #2. (B) The target sequence of TALEN for knockout of miR-122 and the genome sequence of the miR-122 allele in Huh7-122KO cells. Mutations in the miR-122 allele with an 11 nt or a 21 nt deletion in the Huh7-122KO#1 clones or a 2 nt or an 8 nt deletion in the Huh7-122KO#2 clones were identified. (C) Detection of miR-122 expression by Northern blot (top panel) and qRT-PCR (bottom). Total RNA was extracted from each cell and the relative expression of miR-122 was determined by qRT-PCR by using U6 snRNA as an internal control. (D) miR-122 activity in miR-122-knockout Huh7 cells. pmirGLO vectors carrying the complementary sequence of miR-122 under the luciferase gene were transfected into Huh7-122KO and Huh7-122KOR cells. At 48 h post-transfection, the luciferase activity was determined. The data are representative of three independent experiments. Error bars indicate the standard deviation of the mean and asterisks indicate significant differences (**P < 0.01) versus the results for the control. (TIF) [file ppat.1006374.s001.tif]

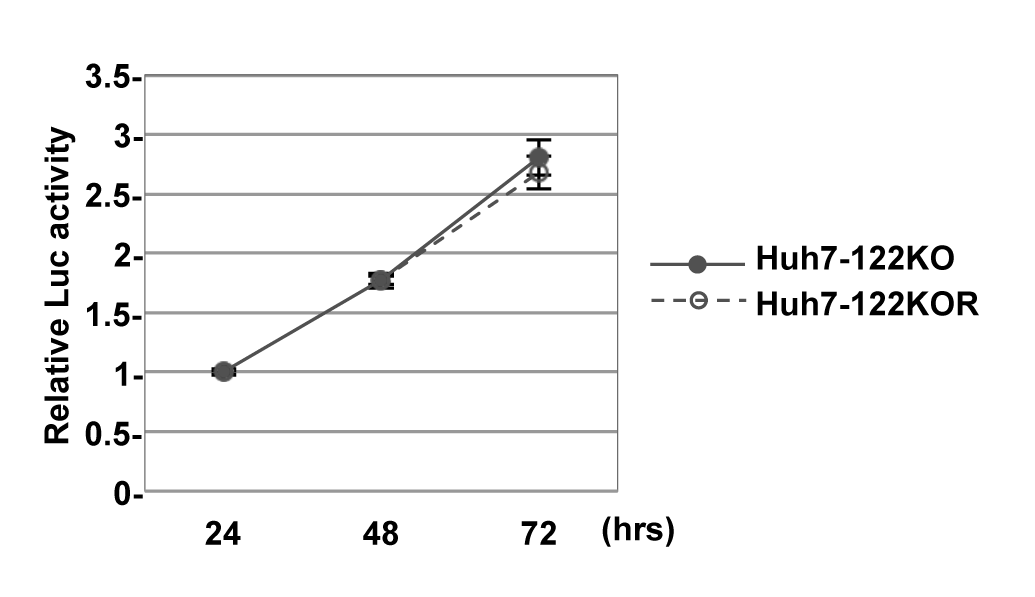

Supplement: S2 Fig — The effect of miR-122 knockout on cell growth was determined by using a Cell Titer-Glo Luminescent Cell Viability Assay. Equal amounts of Huh7-122KO#1 and Huh7-122KOR#1 cells were seeded and RLU were determined at 24, 48, and 72 h post-seeding. (TIF) [file ppat.1006374.s002.tif]

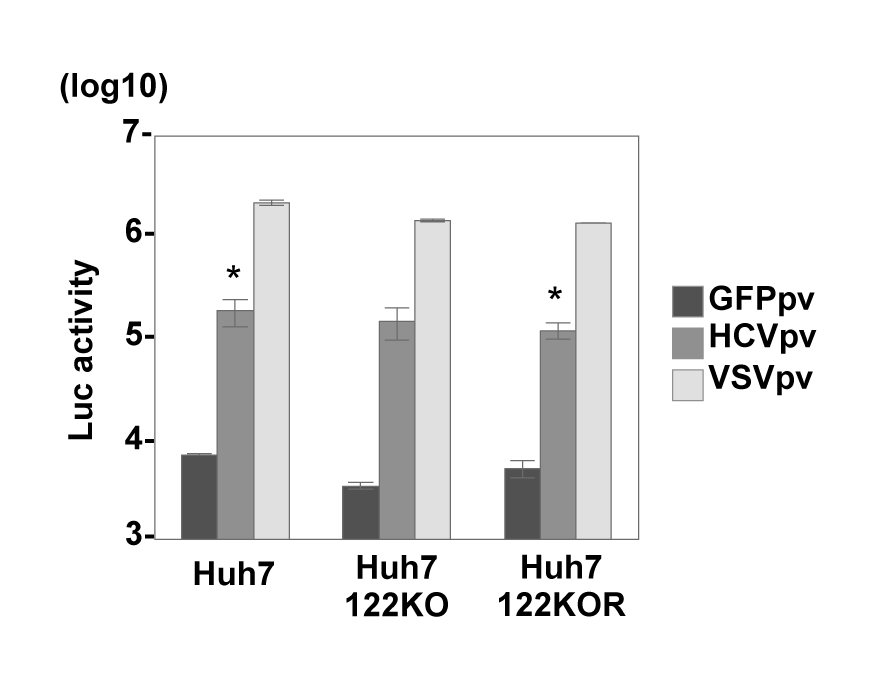

Supplement: S3 Fig — Entry of pseudotyped VSVs bearing no envelope proteins or the HCV and VSV envelope proteins, GFPpv, HCVpv, and VSVpv, respectively, into Huh7, Huh7-122KO, and Huh7-122KOR cells. Luciferase activity was determined at 24 h post-infection. (TIF) [file ppat.1006374.s003.tif]

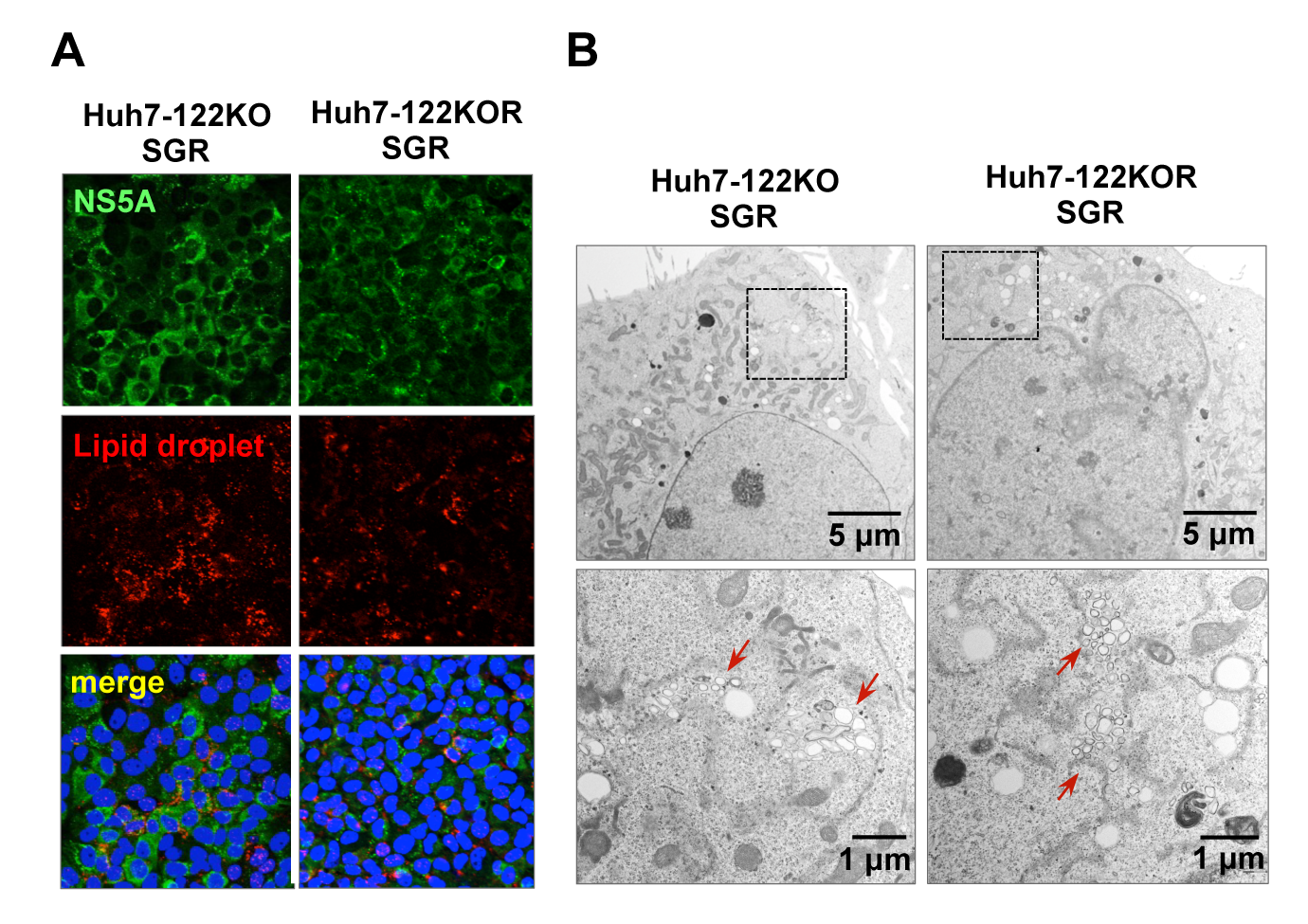

Supplement: S4 Fig — (A) Huh7-122KO-SGR and Huh7-122KOR-SGR cells were fixed with 4% PFA and stained with anti-NS5A antibody (green) and BODIPY for lipid droplets (red). Cell nuclei were stained with DAPI (blue). (B) Electron microscopy of Huh7-122KO-SGR and Huh7-122KOR-SGR cells. The boxes in the lower panels were magnified and the red arrows indicate membranous web-like structures. (TIF) [file ppat.1006374.s004.tif]

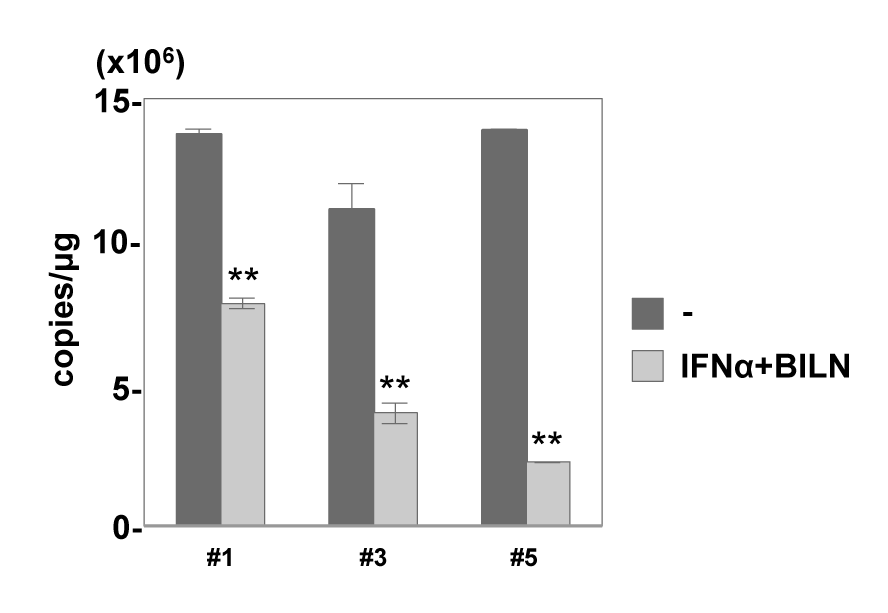

Supplement: S5 Fig — Intracellular HCV-RNA in Huh7-122KO-SGR #1, #3 or #5 cells treated with a combination of 100 IU/ml of IFN-α and 200 nM of the NS3-4A protease inhibitor BILN was quantified by qRT-PCR at 36 hpi. Error bars indicate the standard deviation of the mean and asterisks indicate significant differences (**P < 0.01) versus the results for the control. (TIF) [file ppat.1006374.s005.tif]

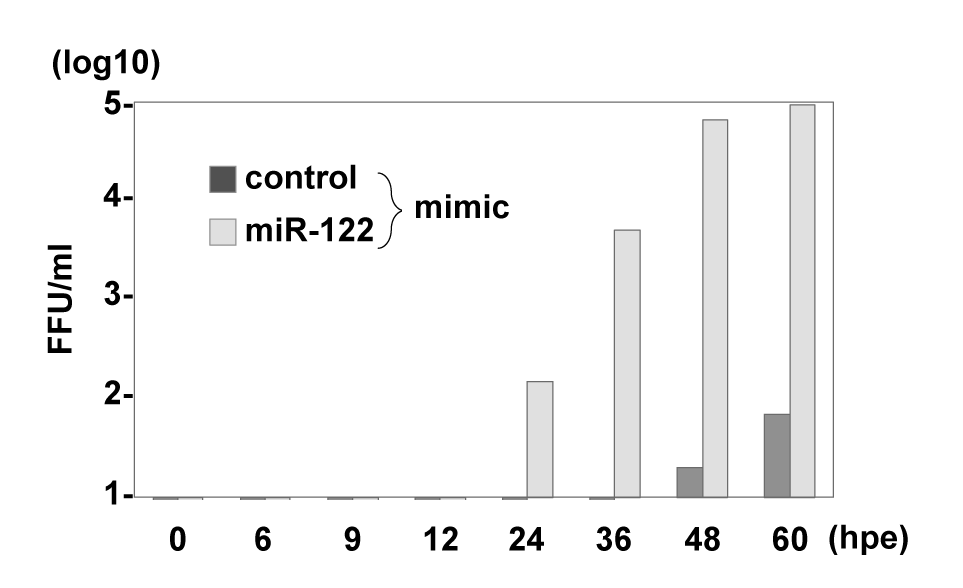

Supplement: S6 Fig — Full-genomic HCV-RNA of the JFH1 strain was electroporated into Huh7-122KO cells together with either the control- or miR-122-mimic, and then the infectious titers in the culture supernatants were determined at 3, 6, 9, 12, 24, 36, 48, and 60 h post-electroporation (hpe). (TIF) [file ppat.1006374.s006.tif]

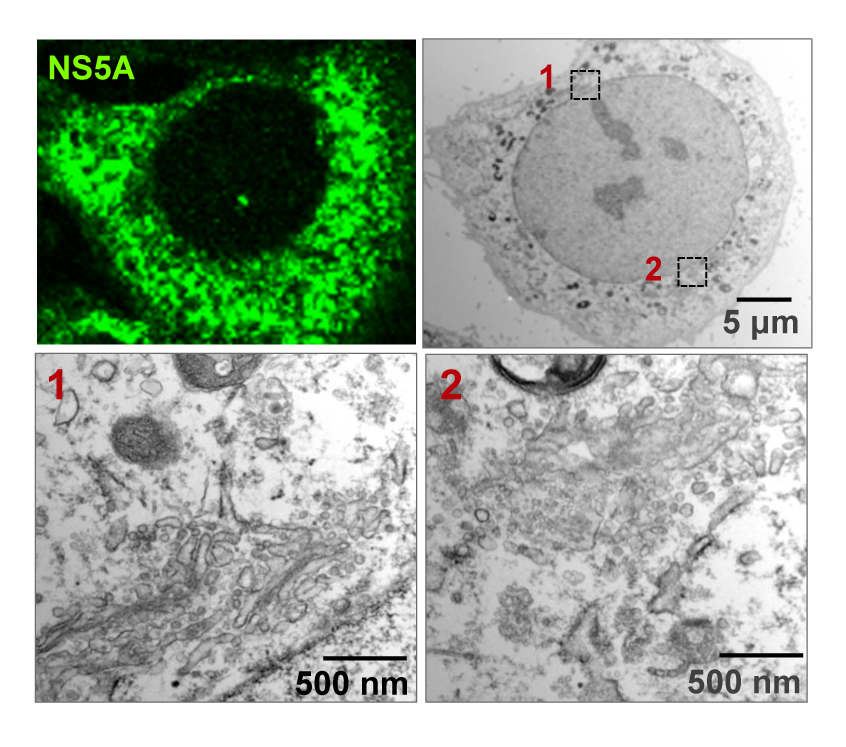

Supplement: S7 Fig — HCV NS5A in Huh7-122KO cells was observed by the FM-EM method. The boxes (1 and 2) in the right top panel were magnified (bottom), respectively. (TIF) [file ppat.1006374.s007.tif]

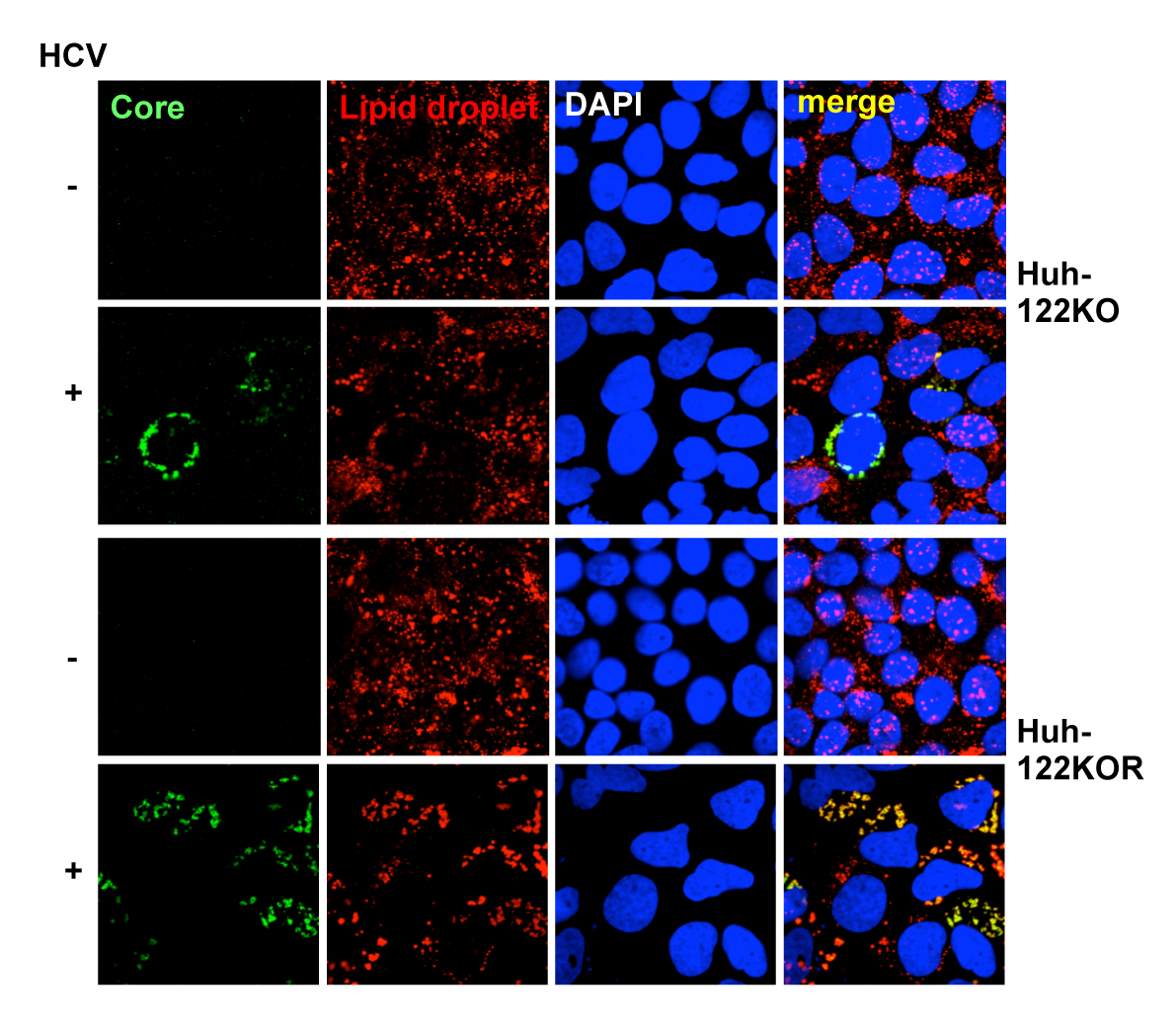

Supplement: S8 Fig — Huh7-122KO and Huh7-122KOR cells infected with HCV and those mock-infected were fixed at 72 hpi and stained with antibodies to core protein (green) and BODIPY for lipid droplets (red). Cell nuclei were stained with DAPI (blue). (TIF) [file ppat.1006374.s008.tif]

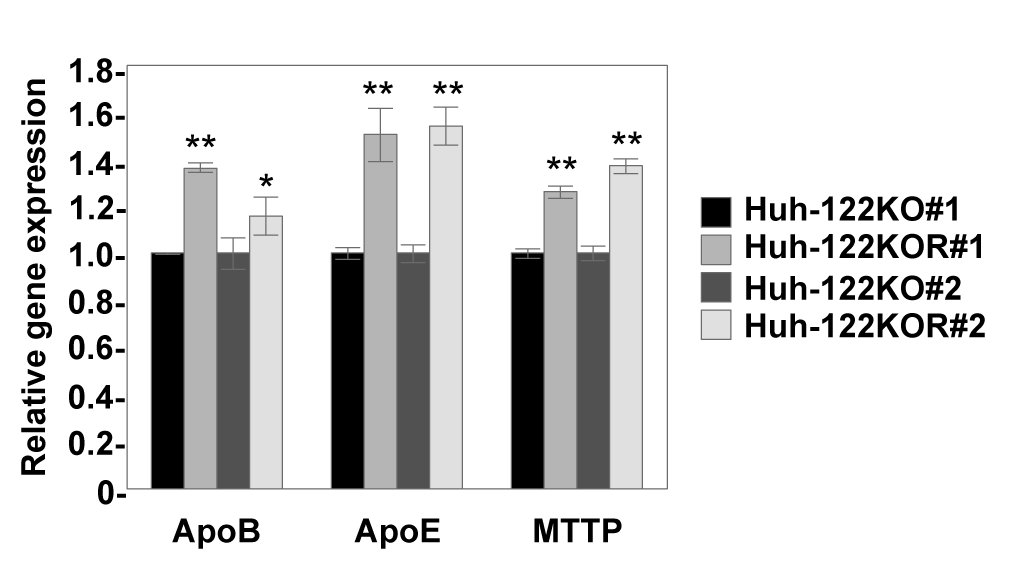

Supplement: S9 Fig — The expression levels of apoE, apoB and MTTP in Huh7-122KO and Huh7-122KOR cells were analyzed by qRT-PCR. Error bars indicate the standard deviation of the mean and asterisks indicate significant differences (*P < 0.05; **P < 0.01) versus the results for the control. (TIF) [file ppat.1006374.s009.tif]

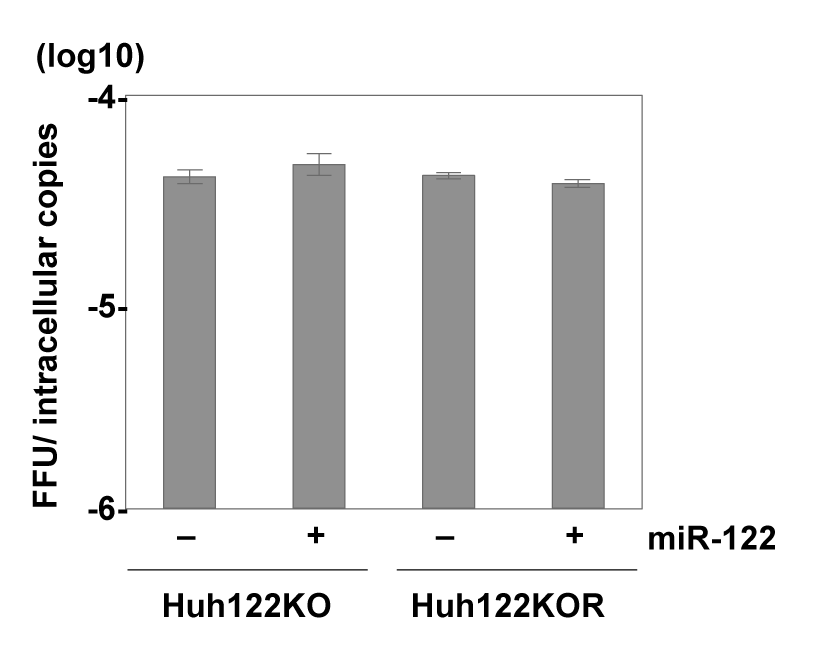

Supplement: S10 Fig — Specific infectivity (infectious titers/intracellular RNA copies) was calculated at 72 h post-infection. (TIF) [file ppat.1006374.s010.tif]

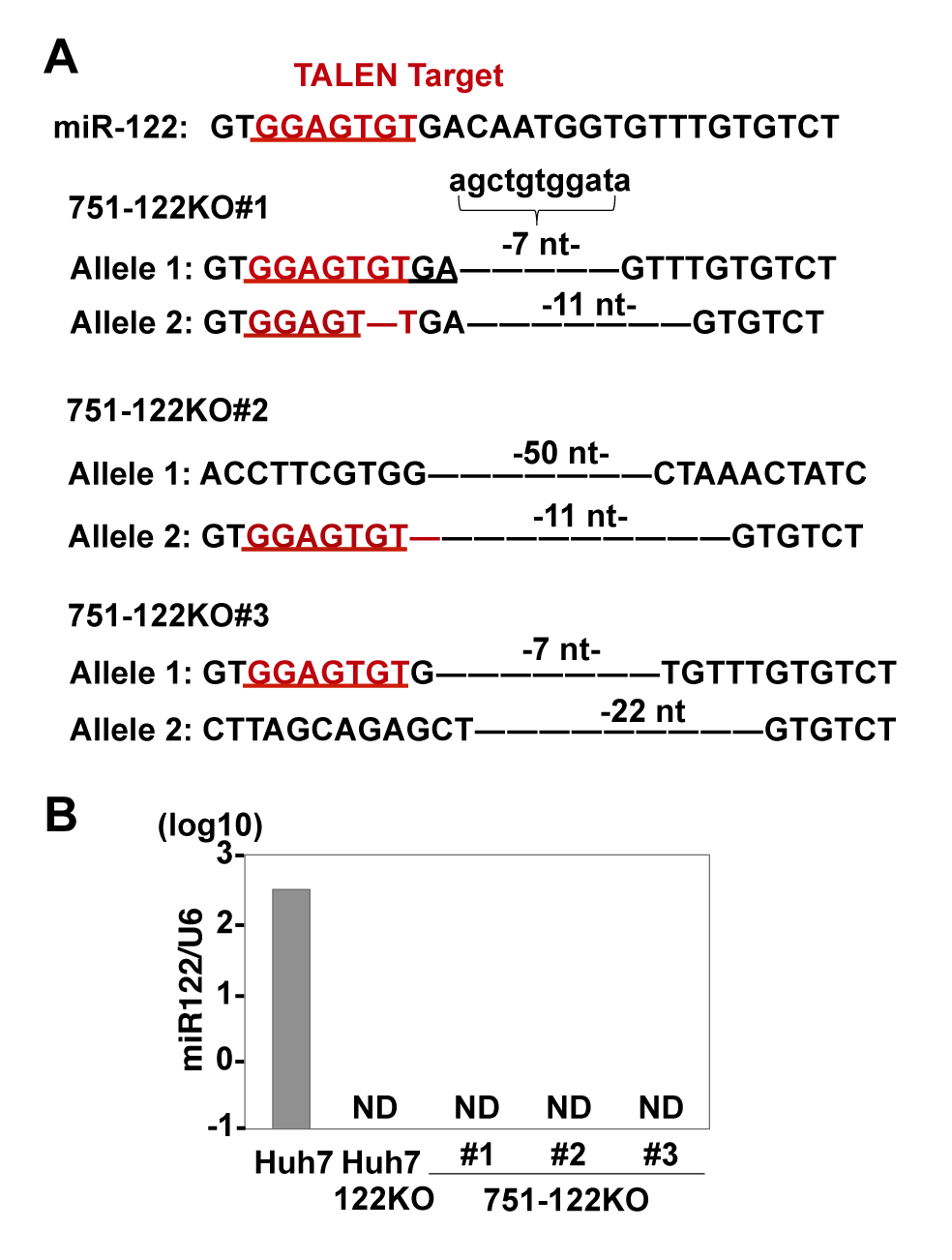

Supplement: S11 Fig — (A) Target sequence of TALEN for knockout of miR-122 and genome sequence of the miR-122 allele in 751-122KO cells. A 10 nt insertion into a 7 nt deletion and an 11 nt deletion, 50 nt and 11 nt deletions, and 7 nt and 22 nt deletions in the miR-122 allele were observed in 751-122KO#1, 751-122KO#2, and 751-122KO#3 cells, respectively. (B) The relative expression of miR-122 was determined by qRT-PCR. (TIF) [file ppat.1006374.s011.tif]

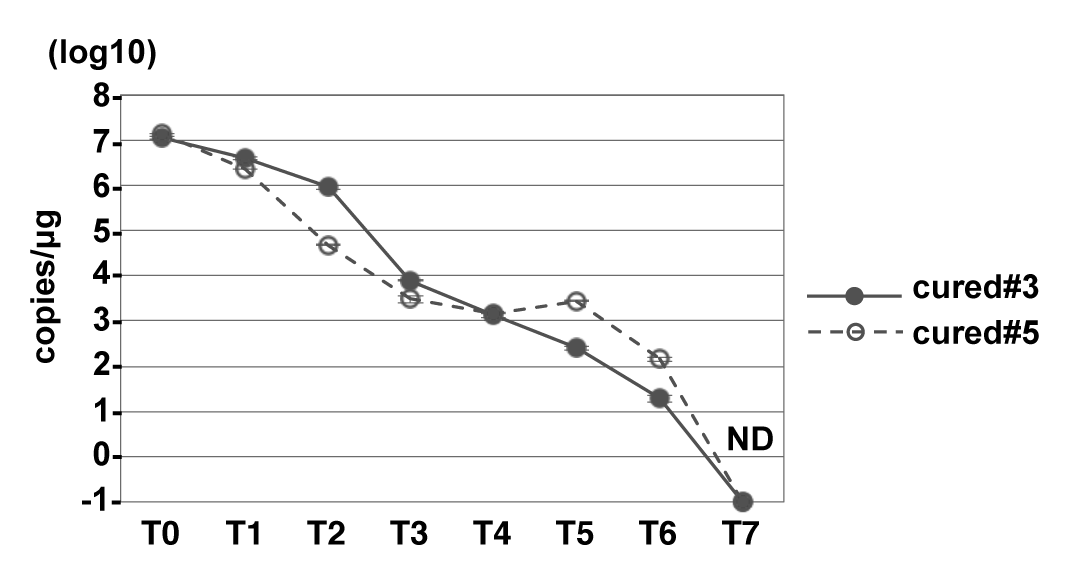

Supplement: S12 Fig — Elimination of HCV-RNA from Huh7-122KO#2-derived JFH-SGR cells. Two clones derived from Huh7-122KO-SGR cells (#3 and #5) were treated with a combination of 100 IU/ml of IFN-α and 200 nM of BILN to eliminate the HCV genome. The intracellular HCV-RNA level at each treatment (every 3 or 4 days) was determined by qRT-PCR. (TIF) [file ppat.1006374.s012.tif]

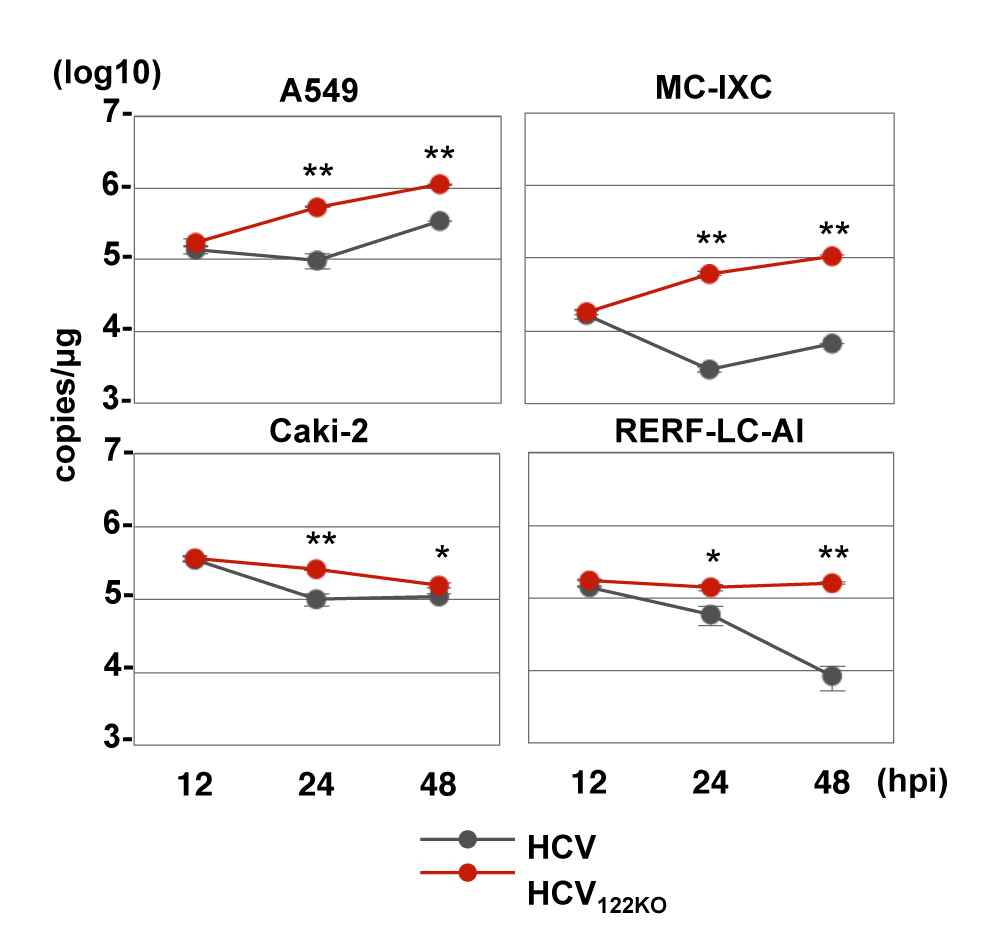

Supplement: S13 Fig — HCV (black circles) and HCV122KO (red circles) were inoculated into non-hepatic A549, Caki-2, MC-IXC and RERF-LC-AI cells at an MOI of 1 and intracellular HCV-RNA was determined by qRT-PCR at the indicated time points. Error bars indicate the standard deviation of the mean and asterisks indicate significant differences (*P < 0.05; **P < 0.01) versus the results for the control. (TIF) [file ppat.1006374.s013.tif]

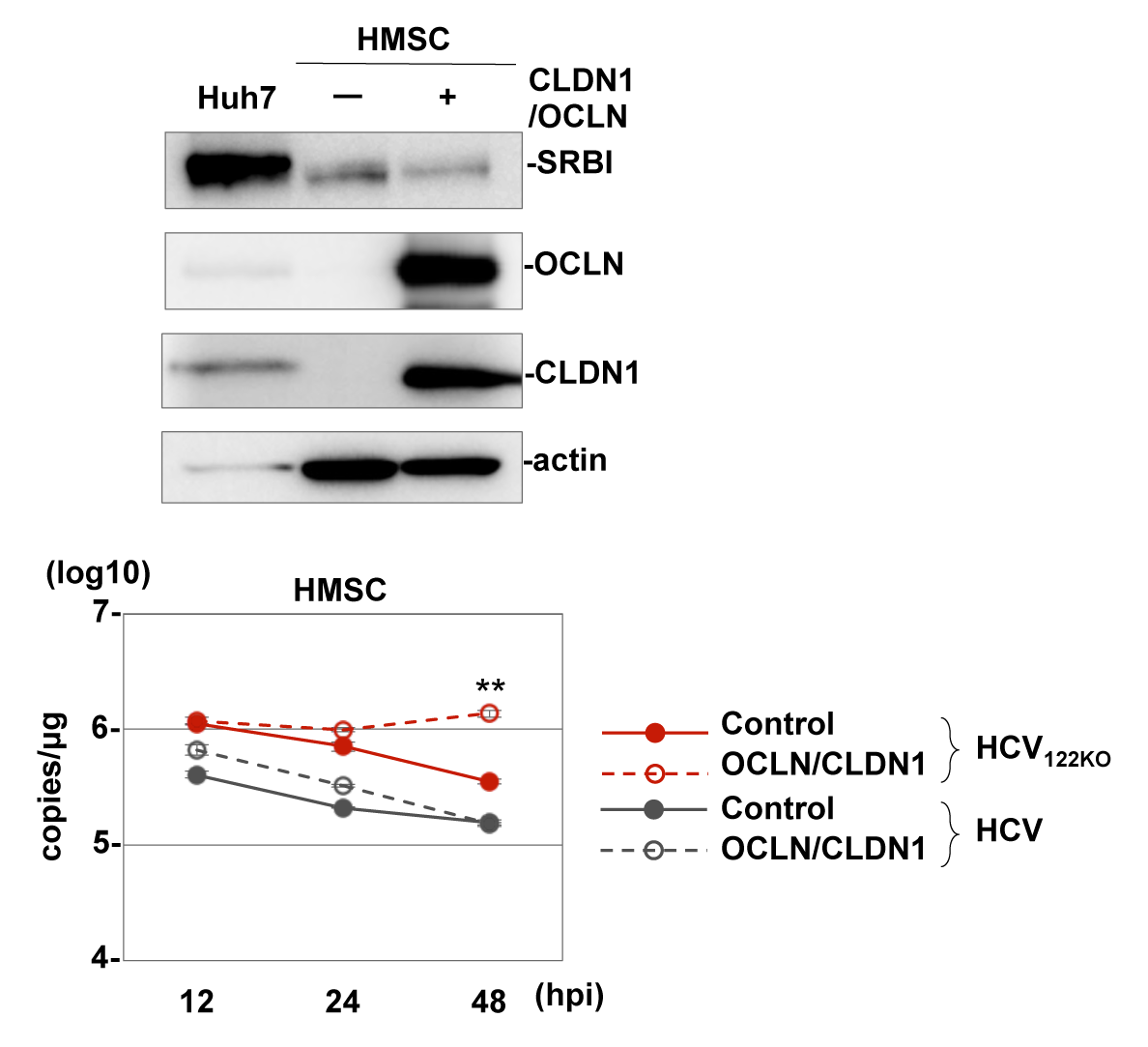

Supplement: S14 Fig — (A) Immunoblotting of SRBI, OCLN, CLDN1 and β-actin in primary HMSC cells exogenously expressing CLDN1 and OCLN. (B) HMSC cells and those expressing CLDN1 and OCLN were infected with either HCV or HCV122KO and intracellular HCV-RNA was determined at 12, 24 and 48 hpi. Error bars indicate the standard deviation of the mean and asterisks indicate significant differences (**P < 0.01) versus the results for the control. (TIF) [file ppat.1006374.s014.tif]

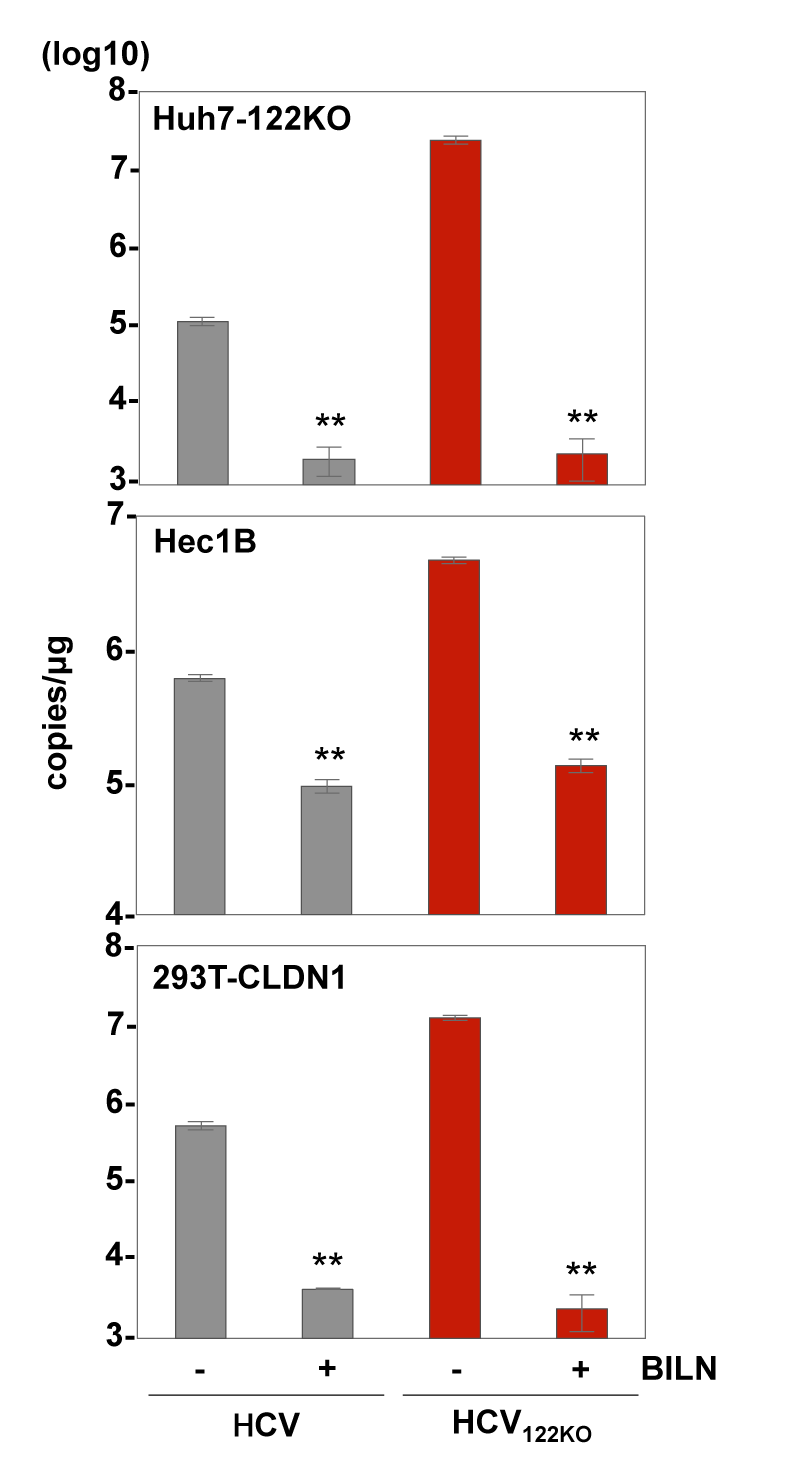

Supplement: S15 Fig — Intracellular HCV-RNA in Huh7-122KO (top), Hec1B (middle) and 293T-CLDN1 cells (bottom) infected with either HCV or HCV122KO were treated with NS3-4A protease inhibitor BILN, and then the intracellular HCV-RNA level was determined by qRT-PCR at 36 hpi. Error bars indicate the standard deviation of the mean and asterisks indicate significant differences (**P < 0.01) versus the results for the control. (TIF) [file ppat.1006374.s015.tif]

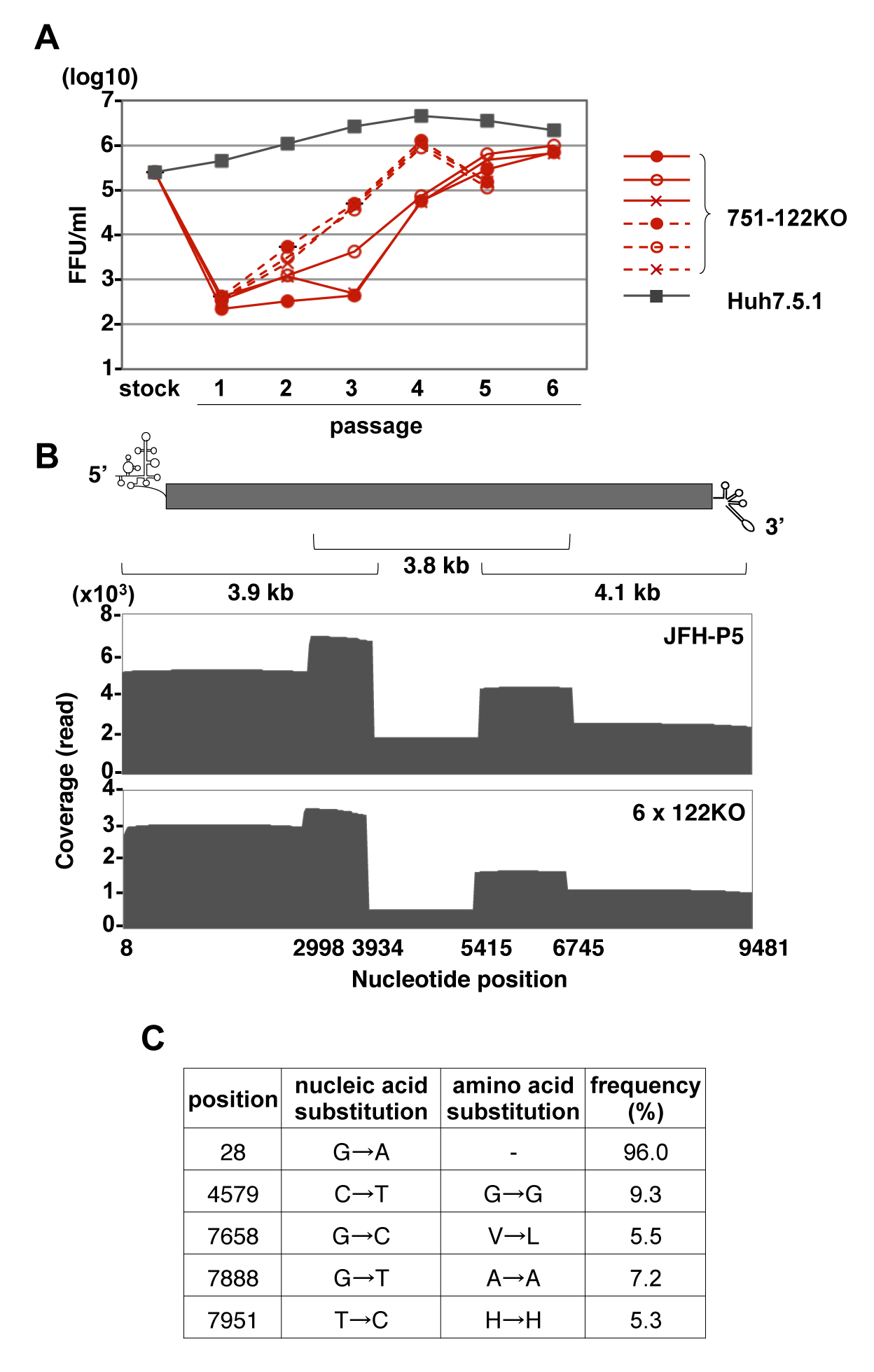

Supplement: S16 Fig — (A) 751-122KO#1 and #2 cells (each from three independent cultures) or Huh7.5.1 cells were infected with HCV at an moi of 10, the culture supernatants were collected after serial passages, and the infectious titers in the supernatants were determined by plaque assay. (B, C) Three fragments amplified from RNA purified from the culture supernatants after 5 passages in Huh7.5.1 cells (JFH-P5) or a mixture of those after 5 and 4 passages in 751-122KO#1 and 751-122KO#2 cells, respectively (6 x 122KO), were sequenced by deep sequencing analysis. The read coverage frequency (B) and the substitutions detected in 6 x 122KO (5% cut-off) (C) are shown. (TIF) [file ppat.1006374.s016.tif]

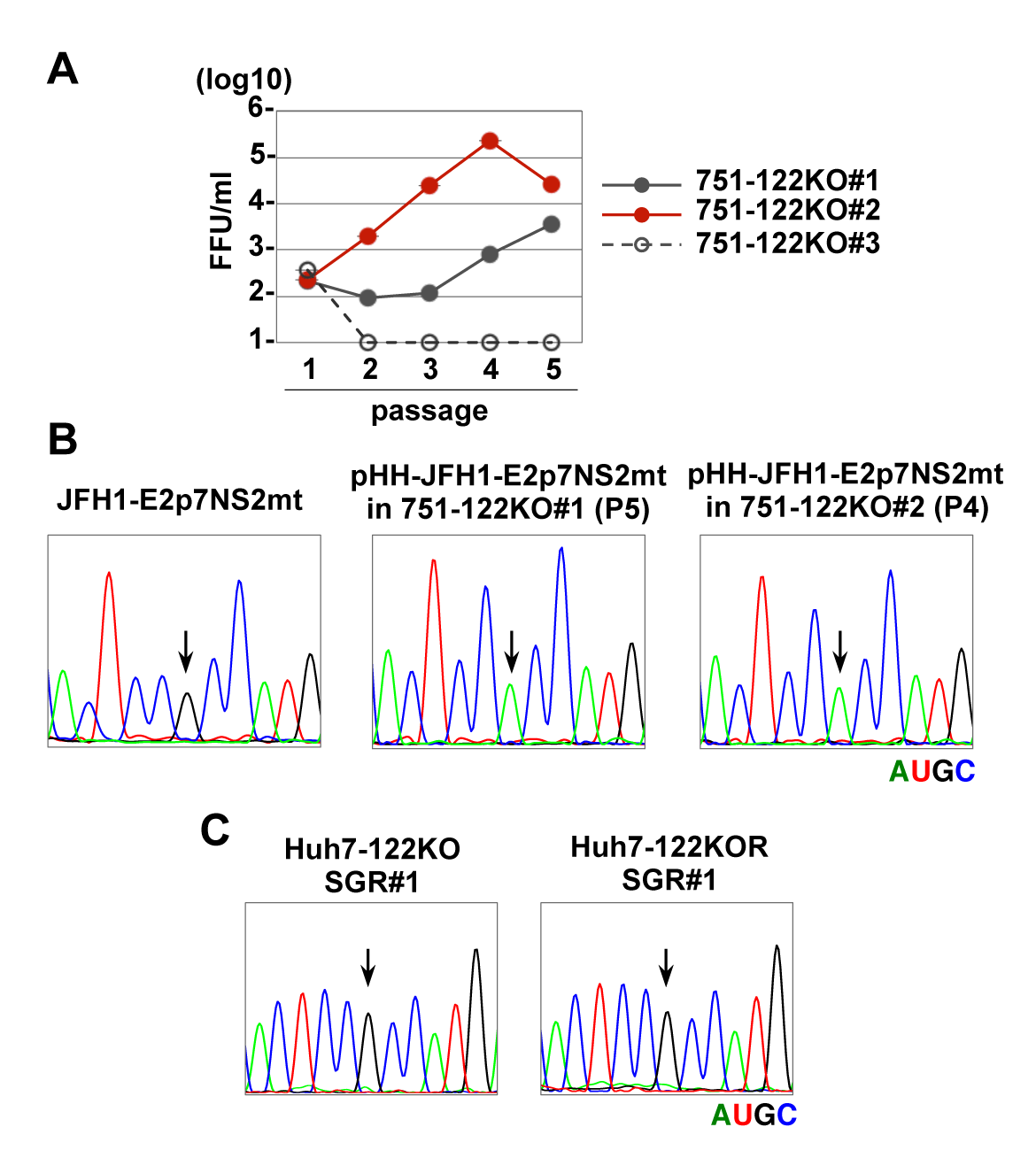

Supplement: S17 Fig — (A) pHH-JFH1-E2p7NS2mt was transfected into 751-122KO (#1, #2 and #3) cells, the culture supernatants were collected after serial passages, and infectious titers in the supernatants were determined by plaque assay. RNA purified from the culture supernatants after 5 and 4 passages in 751-122KO#1 and 751-122KO#2 cells, respectively (B), or Huh7-122KO-SGR#1 and Huh7-122KOR-SGR#1 cells (C) were sequenced. Arrows indicate the position of nt28 in the 5’UTR of JFH1-E2p7NS2mt and isolated viral RNA. Each RNA base is shown as a colored peak: A, green; U, red; G, black; and C, blue. (TIF) [file ppat.1006374.s017.tif]

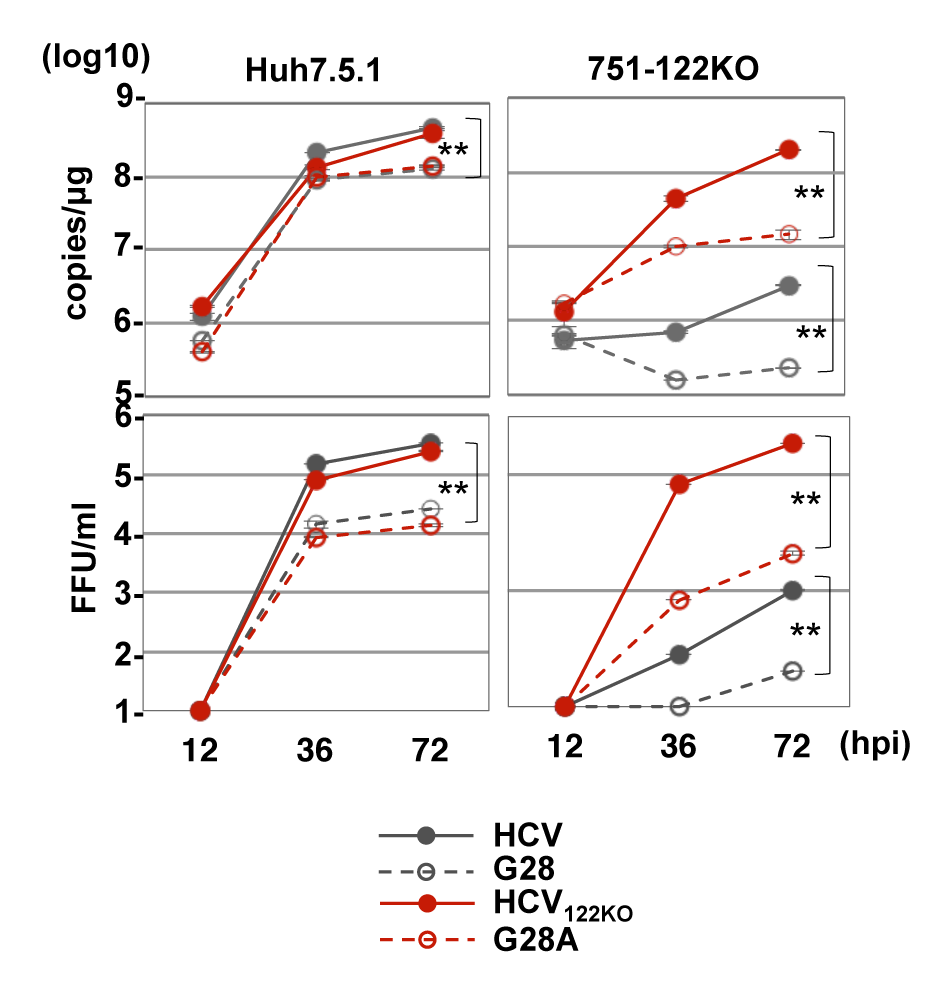

Supplement: S18 Fig — pJFH1-E2p7NS2mt or pJFH1-E2p7NS2mt-G28A RNA was electroporated into Huh7.5.1 cells, and the culture supernatants were collected at 4 dpi as G28 virus or G28A virus. Huh7.5.1 and 751-122KO#1 cells were infected with either G28 virus, G28A virus, HCV or HCV122KO at an MOI of 1 and intracellular HCV-RNA and infectious titers in the supernatants were determined by qRT-PCR and focus formation assay, respectively, at the indicated time points. Error bars indicate the standard deviation of the mean and asterisks indicate significant differences (**P < 0.01) versus the results for the control. (TIF) [file ppat.1006374.s018.tif]

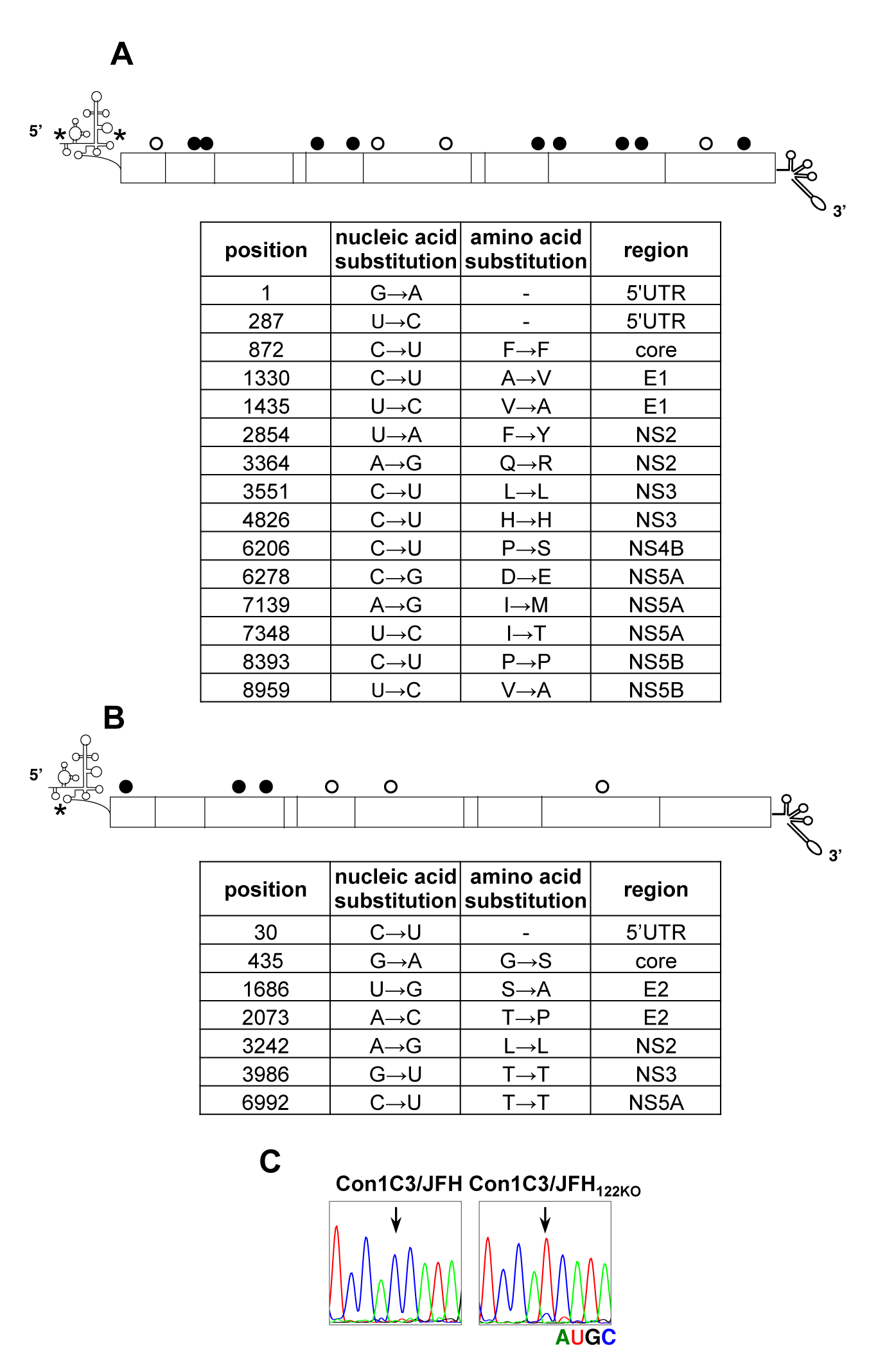

Supplement: S19 Fig — Three amplified fragments of cDNA derived from Con1C3/JFH (A) or Con1C3/JFH122KO (B) were sequenced by direct sequencing analysis. Black circles (non-synonymous substitution), white circles (synonymous substitution) and asterisks (single nucleotide mutation in 5’UTR) are shown at the position of each mutation on the diagram of the HCV-RNA structure. Only additional mutations were shown in Con1C3/JFH122KO (B) compared to Con1C3/JFH. (C) RNA purified from Con1C3/JFH or Con1C3/JFH122KO were sequenced, respectively. Arrows indicate the position of nt30 in the 5’UTR of isolated viral RNA. Each RNA base is shown as a colored peak: A, green; U, red; G, black; and C, blue. (TIF) [file ppat.1006374.s019.tif]

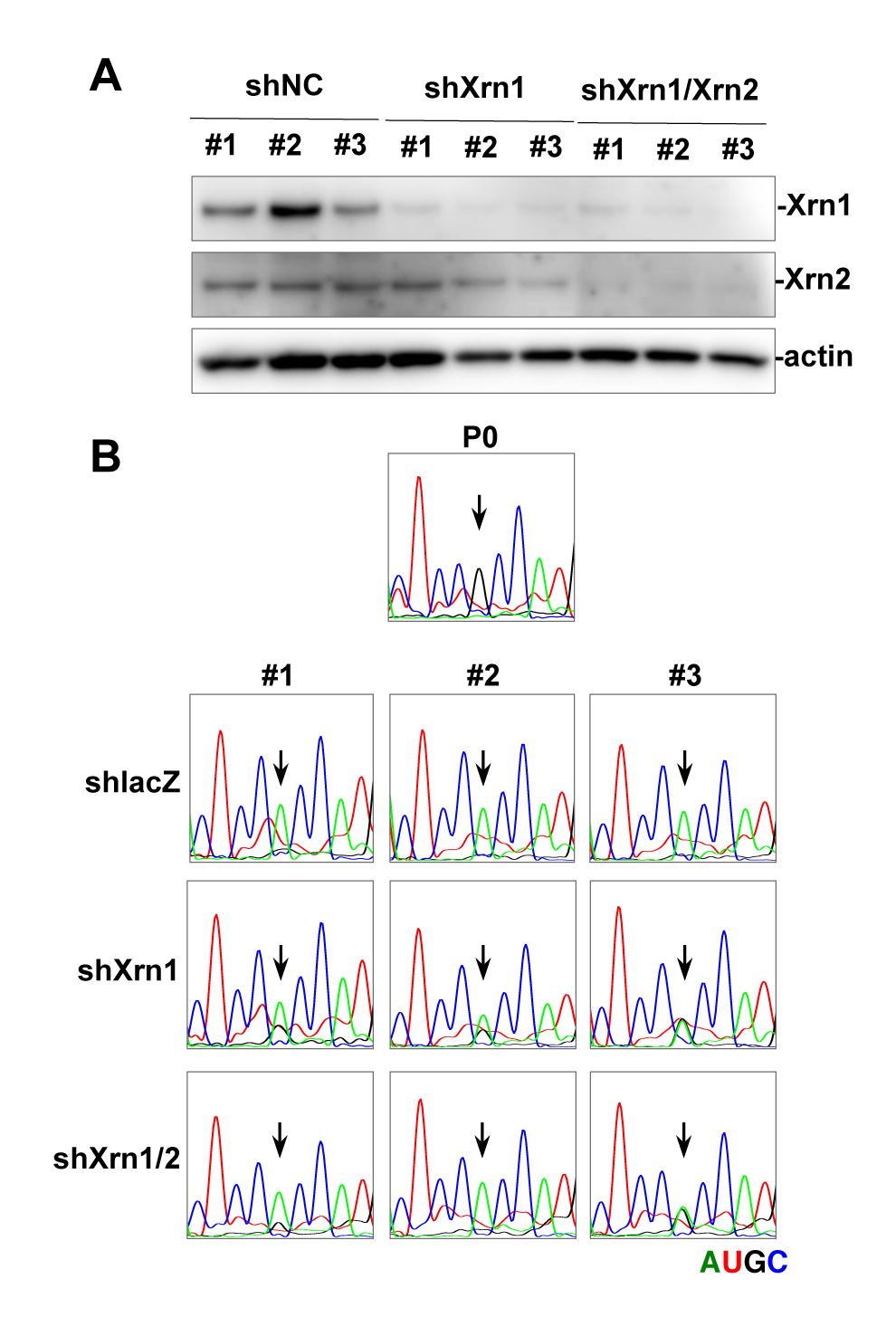

Supplement: S20 Fig — (A) Establishment of Xrn1 or Xrn1/Xrn2 stable-knockdown miR-122 KO Huh7.5.1 cells. The results of immunoblotting of Xrn1, Xrn2 or β-actin from three clones each of 751-122KO-shlacZ, 751-122KO-shXrn1 or 751-122KO-shXrn1/Xrn2 cells (#1~#3) were shown. (B) Direct sequencing analysis of adapted viruses independently isolated in each of 751-122KO-shlacZ, 751-122KO-shXrn1 or 751-122KO-shXrn1/Xrn2 cells at passages 0 (virus stock) and 4 are shown. Arrows indicate the position of nt28 in the 5’UTR of HCV. Each RNA base is shown as a colored peak: A, green; U, red; G, black; and C, blue. (TIF) [file ppat.1006374.s020.tif]

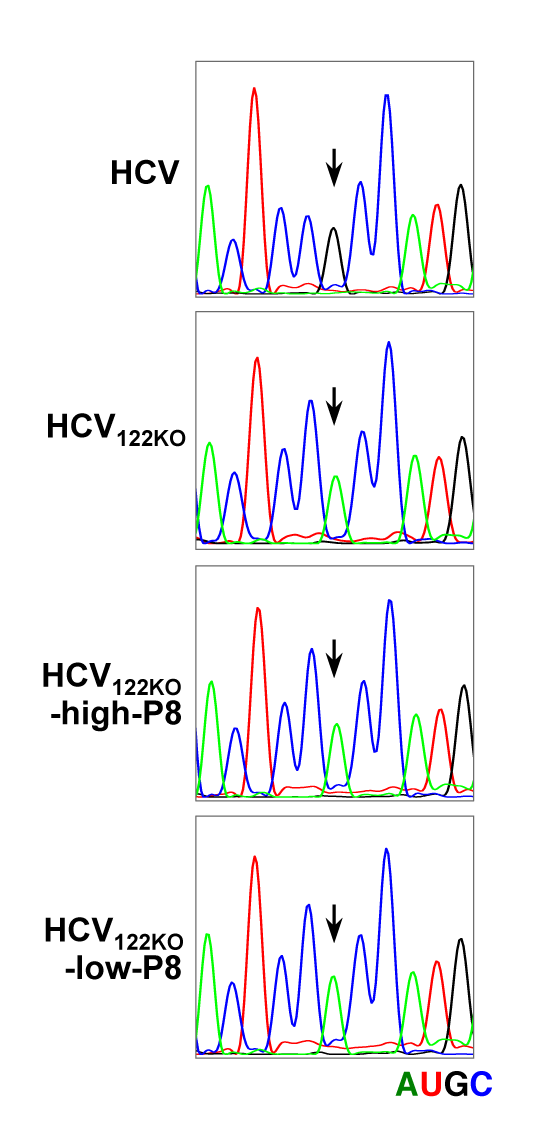

Supplement: S21 Fig — Huh7.5.1 cells were infected with HCV122KO at a high or low titer and collected after eight serial passages. RNA purified from the culture supernatants was sequenced. Arrows indicate the position of nt28 in the 5’UTR of each isolated viral RNA. Each RNA base is shown as a colored peak: A, green; U, red; G, black; and C, blue. (TIF) [file ppat.1006374.s021.tif]

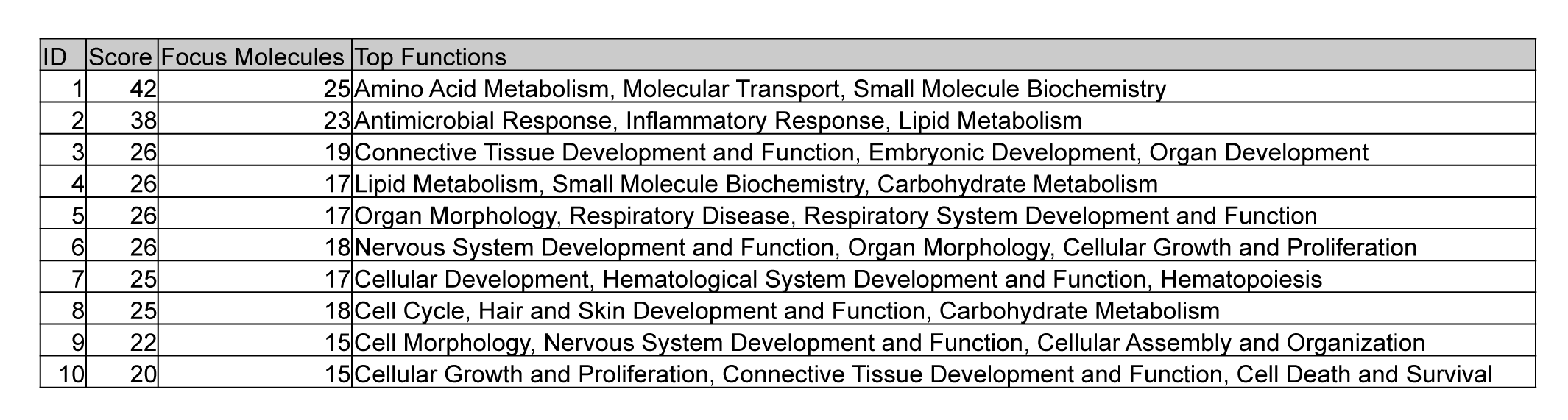

Supplement: S1 Table — The top 10 predicted pathways generated by IPA from the change of gene expression by miR-122 knockout. (TIF) [file ppat.1006374.s022.tif]

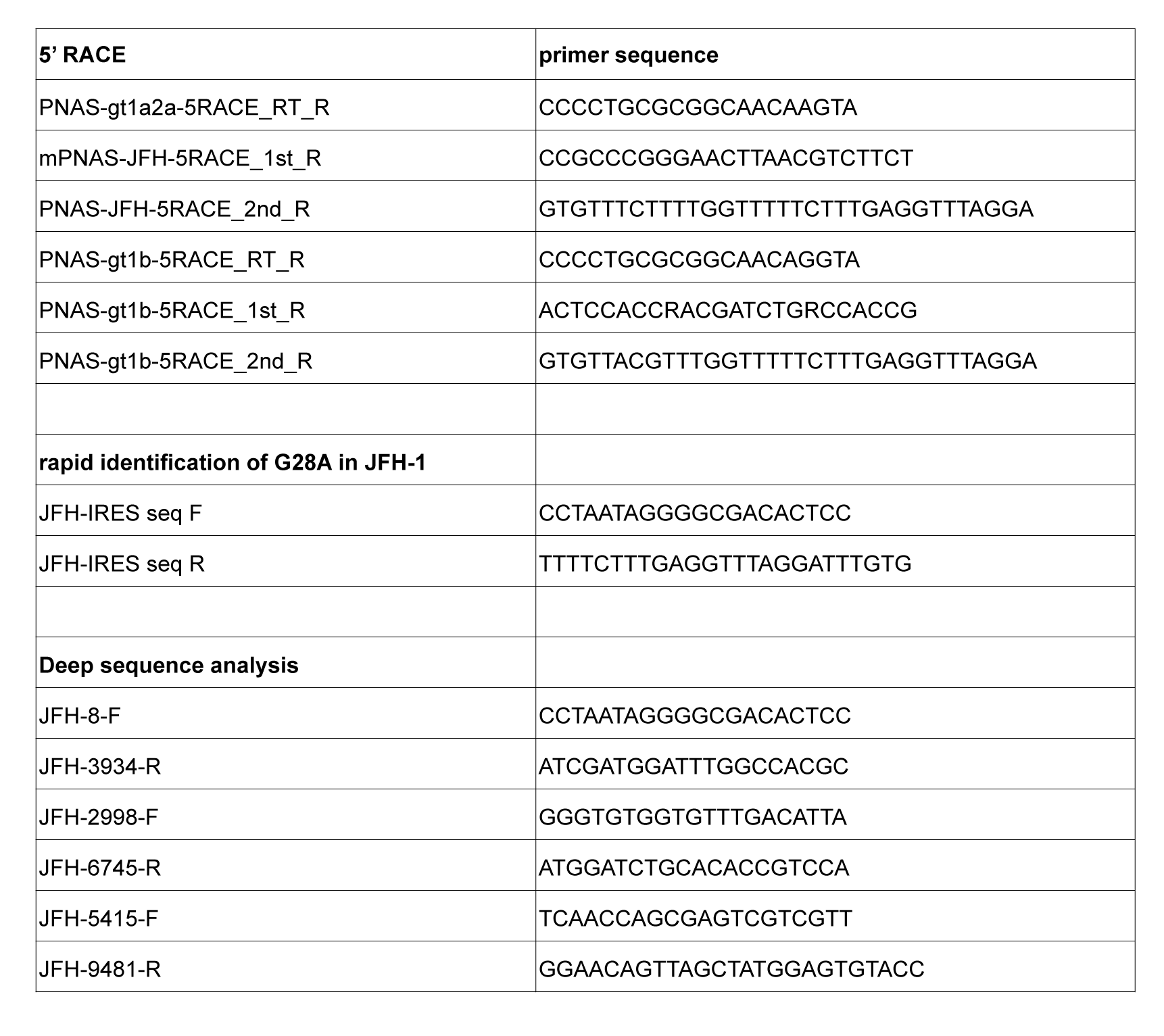

Supplement: S2 Table — (TIF) [file ppat.1006374.s023.tif]
